# A Nature-Based Intervention and Mental Health of Schoolchildren: A Cluster Randomized Clinical Trial

**DOI:** 10.1001/jamanetworkopen.2024.44824

**Published:** 2024-11-15

**Authors:** Tianna Loose, Julia Fuoco, Catherine Malboeuf-Hurtubise, Jean-Philippe Ayotte-Beaudet, Lise Gauvin, Nicholas Chadi, Isabelle Ouellet-Morin, Benoît Mâsse, Sylvana M. Côté, Marie-Claude Geoffroy

**Affiliations:** 1 Department of Social and Preventive Medicine, School of Public Health, University of Montreal, Montreal, Quebec, Canada; 2 Sainte Justine Hospital (CHU Sainte-Justine) Research Centre, Montreal, Quebec, Canada; 3 Douglas Mental Health University Institute, Montreal, Quebec, Canada; 4 Department of Psychology, Bishop’s University, Sherbrooke, Quebec, Canada; 5 Sherbrooke Hospital Research Centre, Sherbrooke, Quebec, Canada; 6 Department of Preschool and Elementary Education, University of Sherbrooke, Sherbrooke, Quebec, Canada; 7 University of Montreal Hospital Research Centre (Centre de Recherche du Centre Hospitalier de l’Université de Montréal), Montreal, Quebec, Canada; 8 Department of Pediatrics, University of Montreal, Montreal, Quebec, Canada; 9 School of Criminology, University of Montreal, Montreal, Quebec, Canada; 10 Research Center of the Montreal Mental Health University Institute (Institut Universitaire en Santé Mentale de Montréal), Montreal, Quebec, Canada; 11 School of Public Health, University of Montreal, Montreal, Quebec, Canada; 12 Department of Psychiatry and Epidemiology, Biostatistics and Occupational Health, McGill University, Montreal, Quebec, Canada

## Abstract

**Importance:**

Nature-based therapeutic or preventive interventions for mental health are increasingly popular, but their effectiveness for improving mental health is not well documented.

**Objective:**

To investigate the effectiveness of the Open Sky School Program (École à Ciel Ouvert), a 12-week nature-based intervention for elementary schoolchildren in grades 5 and 6, for reducing mental health symptoms.

**Design, Setting, and Participants:**

This 2-arm, cluster randomized clinical trial was conducted from February 27 to June 16, 2023, in French-language elementary schools in Quebec, Canada, with green space within 1 km. Participants were teachers and students in grades 5 and 6.

**Intervention:**

In the intervention group, for 2 hours per week for 12 weeks, classes were taught outdoors in a nearby park or wooded area. Teachers were encouraged to engage students in basic subjects and in 10 or more mental health activities (mindfulness, philosophy, and/or art therapy). Control group schools conducted classes as usual.

**Main Outcomes and Measures:**

The primary outcome was change in student mental health (internalizing and externalizing symptoms, social problems) based on teacher- and student-reported 30-item Social Behavior Questionnaire (SBQ) scores (3-point scale) from baseline to the immediate postintervention follow-up, assessed in per-protocol and intent-to-treat mixed-model analyses. Secondary outcomes were student self-reported changes in depressive symptoms, positive or negative affect, pro-environmental efforts and/or attitudes, and nature connectedness.

**Results:**

A total of 33 schools participated (53 teachers, 1015 students), including 16 schools (25 teachers, 515 students) in the intervention group and 17 schools (28 teachers, 500 students) in the control group. Student mean (SD) age was 10.9 (0.75) years; 507 (50.7%) were girls. Per-protocol and intent-to-treat mixed-model analyses showed no differences in mental health symptom change between groups; for example, the adjusted mean difference in SBQ scores between the intervention and control groups for externalizing symptoms was −0.04 (95% CI, −0.13 to 0.04) in the intent-to-treat analysis and −0.06 (95% CI, −0.16 to 0.04) in the per-protocol analysis. Post hoc analyses revealed low mental health symptoms at baseline, with low variability. Slightly greater reductions in symptoms were observed in the intervention group, but only for children with higher mental health symptoms at baseline (*P* < .05 for interaction). For example, for children with internalizing symptoms 1 SD above the mean at baseline, internalizing symptoms decreased by 0.38 SD (mean change, −0.15; *P* < .001) in the intervention group vs the control group.

**Conclusions and Relevance:**

In this large cluster randomized clinical trial in daily-life elementary school settings, 12 weeks of classes in green space for 2 hours per week did not reduce mental health symptoms in students aged 10 to 12 years in either the per-protocol or the intent-to-treat analysis. However, this low-cost, safe outdoor intervention may provide unmeasured or longer-term benefits for children with higher risk of mental health symptoms.

**Trial Registration:**

ClinicalTrials.gov Identifier: NCT05662436

## Key Points

**Question:**

Does a 12-week, nature-based intervention in grades 5 and 6 in elementary schools reduce mental health symptoms in children?

**Findings:**

In this cluster randomized clinical trial, per-protocol and intent-to-treat analyses including 33 schools, 53 teachers, and 1015 students showed no reduction in mental health symptoms immediately after the intervention.

**Meaning:**

The findings of this study suggest that implementing nature-based interventions within the school setting does not reduce children’s mental health symptoms.

## Teaser

This cluster randomized clinical trial investigates the effectiveness of a nature-based outdoor educational intervention for reducing mental health symptoms among elementary school students in grades 5 and 6.

## Introduction

Observational studies suggest that time outdoors (ie, in nature) benefits the mental health of adults and children.^1,2,3,4^ A recent systematic review of 29 observational studies indicated an improvement in children’s emotional and behavioral problems, whether social (eg, victimization, low prosocial behaviors), externalizing (eg, hyperactivity), or internalizing (eg, anxiety, depression).^4^

Various initiatives have promoted outdoor play in natural environments, such as the American Academy of Pediatrics and the Canadian Paediatric Society recommendations for child development and health.^5,6^ Health care professionals in many countries are recommending time in nature to alleviate mental health symptoms.^7^ It is therefore important to better understand under which circumstances nature improves mental health, for whom the effects would be most beneficial, and which aspects of mental health would be best targeted.

Schools can provide students with opportunities to be in contact with nature,^8^ a practice encouraged in many countries, including Canada.^9,10^ Evidence of effectiveness remains limited to observational studies, with mixed results.^4^ To our knowledge, only 2 nonrandomized controlled trials have tested the association of weekly outdoor education over the school year with reducing internalizing symptoms, externalizing symptoms, and social problems in children.^11,12^ One of these studies reported a reduction in internalizing and externalizing symptoms among boys but not girls.^12^ The second reported an increase in prosocial behaviors in all students and a reduction in hyperactivity and peer problems among socioeconomically disadvantaged students.^11^ However, neither study required the out-of-classroom sessions to be in green space. Furthermore, to our knowledge, the effectiveness of education in nature for reducing mental health symptoms has never been tested with a robust randomized clinical design.

We designed a nature-based intervention, the Open Sky School Program, consisting of outdoor classes in a park or wooded area for 2 hours per week for 12 weeks. We hypothesized that exposure to green space would lead to reductions in mental health symptoms. Using a cluster randomized clinical trial design in grades 5 and 6 in elementary schools, we explored potential moderators of effectiveness, including student sex^12,13^ and disability or special needs status,^14^ amount of neighborhood green space,^15^ a socioeconomic disadvantage indicator^11^ of each school, and prior outdoor teaching experience of the teacher.^16^ Post hoc analyses examined effectiveness as a function of symptom level at baseline.^17^

## Methods

### Study Design, Setting, and Participants

We conducted a 2-arm, cluster randomized clinical trial (NCT05662436) from February 27 to June 16, 2023, throughout Quebec, Canada, to examine the effectiveness of the Open Sky School Program (École à Ciel Ouvert) in grades 5 and 6 in French-language elementary schools, as reported by teachers and students. Schools were eligible if located within 1 km of a park or wooded area. The study was approved by the research ethics board of the Montreal West Island Integrated University Health and Social Services Centre. Participation of classes within participating schools was voluntary; teachers provided written informed consent. While students could not opt out of class activities, parents provided written informed consent, and students provided assent, for assessments. The protocol was peer reviewed and published,^18^ and the original protocol is provided in Supplement 1. We followed the Consolidated Standards of Reporting Trials (CONSORT) guideline,^19^ including reporting of protocol deviations (eMethods 1 in Supplement 2).

Eligible schools included those participating in a 2021 COVID-19 study by our group in grade 4 classes.^20,21^ To enhance sample size, we also advertised on social media. Furthermore, teachers received CAD$100 for preintervention and postintervention assessments (maximum, CAD$200) (multiply Canadian dollars by 0.73 to convert to US dollars). Two students per class were randomly selected to win a CAD$50 gift card for completion of both preintervention and postintervention questionnaires. Those who completed the 3-month follow-up had a chance to win 1 of 10 CAD$100 gift cards.

### Randomization

Randomization was at the school level to avoid contamination bias; all students at the same school were allocated to the same group. Stratified randomization (1:1 balance between the number of students in the intervention and control groups) was computerized by an independent, blinded statistician not involved in the study. For example, if school 1 had 40 participants and schools 2 and 3 each had 20 participants, school 1 was assigned to group 1 while schools 2 and 3 were assigned to group 2. Teachers were informed of their grouping by email.

### Outcome Measures

Teachers and students completed questionnaires at baseline (T0) and immediately after the intervention (at 12 weeks [T1]). Students completed the secondary measures follow-up 3 months after intervention end (T2). Details are given in eMethods 2 in Supplement 2.

The primary outcome was change in child mental health between baseline and T1 (internalizing symptoms, externalizing symptoms, and social problems), as measured by the adapted 30-item Social Behavior Questionnaire (SBQ)^22,23^ completed by teachers and students. The SBQ evaluates symptom frequency over the past 2 months on a 3-point scale (never or not true = 0, sometimes or somewhat true = 1, and often or very true = 2). We retained a continuous score for each subscale.

Secondary outcomes were changes in mental health, attitudes and behaviors related to the environment, and nature connectedness, as self-reported by students at T0, T1, and T2. Measures over the past 2 weeks included positive and negative affect (20-item Positive and Negative Affect Schedule for Children,^24^ on a 5-point Likert scale with 1 indicating very slightly or not at all and 5, extremely) and depressive symptoms (13-item Children’s Depression Inventory–Short Version; children considered how they were feeling over the past 2 weeks and responded on a 3-point scale, eg, 1 = I hate myself, 2 = I don’t like myself, and 3 = I like myself).^25^ Environmental outcomes included nature connectedness (6-item Nature Connection Index)^26^ and pro-environmental behaviors (eg, recycling) and efforts (eg, volunteering), as measured by 6 items from a previous study.^27^

Potential moderating variables were sex of the child (self-report), disability or special needs status (teacher report), neighborhood green space (density of green vegetation associated with the school postal code, quantified by the Normalized Difference Vegetation Index),^28^ school socioeconomic disadvantage indicator (Quebec Ministry of Education: 1 = most advantaged or private school; 10 = most disadvantaged),^29^ and teacher experience with outdoor education in past 3 years (1 = yes, 0 = no). We also investigated the intervention’s effectiveness as a function of baseline mental health symptoms (at T0).^17^

To indicate adherence, teachers in the intervention group completed a weekly online logbook describing implementation of outdoor activities. Teachers in the control group completed a questionnaire immediately after the intervention (T1), indicating engagement in outdoor education.

### Intervention

An activity tool kit was designed by experts in education and clinical psychology.^30^ Teachers brought their class to a nearby green space for 2 hours per week for 12 weeks (2-hour sessions or two 1-hour sessions on or off school property). Using the tool kit and other teacher-initiated activities, they engaged in basic subjects (language, mathematics, and science) and in mental health improvement activities (mindfulness, philosophy, and art therapy) over the intervention period. Teachers had access to 2 hours per week of virtual optional consultation for the first 3 weeks. Thereafter, team members answered questions by email or videoconference. Details are given in eMethods 3 in Supplement 2.

The control group continued with education as usual. Teachers in the control group were not prohibited from taking their classes outside on their own. Teachers were offered access to the intervention the following year.

### Statistical Analysis

Data were collected using Qualtrics XM software (Qualtrics). We used SPSS, version 20 (IBM Corp) and Python, version 3.11.8 (Python Software Foundation) for statistical analysis. The targeted sample size of 2500 students was designed for 80% power and an α<.05 to detect small effect sizes (0.20) for primary and secondary outcomes.

Descriptive statistics included outcomes at baseline and immediately after the intervention. To test effectiveness, we performed mixed-model analyses, with random effects clustered at the school level. Clustering at the school level allowed for intraclass correlations within each school. Mixed-model analyses were adjusted for confounding variables and mental health assessments at baseline. For instance, for teacher-reported internalizing symptoms as an outcome, we used group (intervention or control) as a between-participant independent factor, baseline internalizing symptoms and other confounders as control variables, and school as a clustered random effect. We reported mean changes, 95% CIs, and *P* values.

We conducted both intent-to-treat and per-protocol analyses. Per-protocol analyses excluded intervention group classrooms that did not engage in 80% of the intervention. For mixed-model analyses for primary outcomes, significance was set at 2-sided *P* <.025 (2/.05) to adjust for co–primary outcomes. For post hoc analyses, significance was set at 2-sided *P* < .05. We explored potential moderators by adding an interaction term to intent-to-treat mixed-model analyses (eg, boys compared with girls). All analyses were conducted with the maximum available sample. We deemed any attrition to be random (eg, children absent at questionnaire completion time or change in teachers between baseline and follow-up).

## Results

### Participants

**Figure 1.**
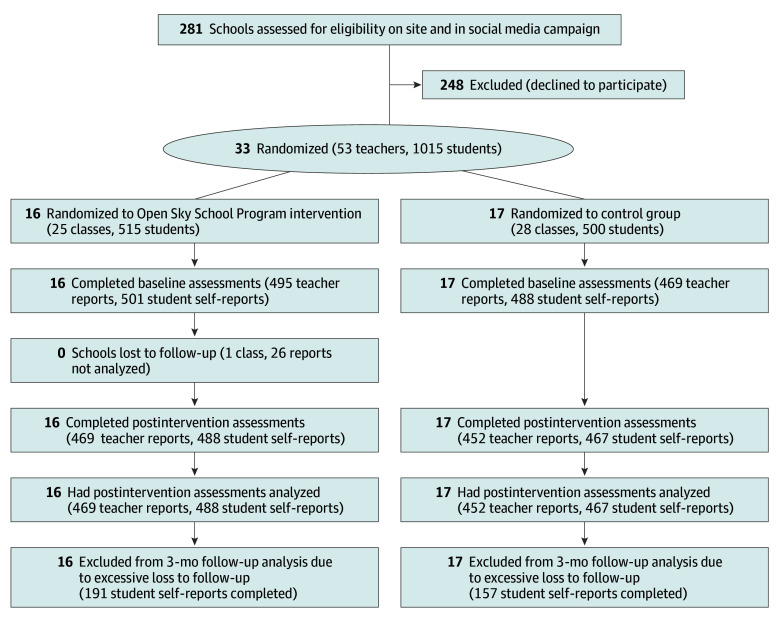
CONSORT Flow Diagram of Study Participants

Of 281 schools approached directly or through social media campaigns, 33 schools participated (11.7%), with 53 grade 5 and 6 teachers and 1015 students agreeing to questionnaire completion. The intervention group included 16 schools with 25 teachers and 515 students, and the control group included 17 schools with 28 teachers and 500 students (Figure 1). Mean (SD) age of student participants was 10.9 (0.75) years, 493 (48.6%) were boys, 507 (50.7%) were girls, and 112 (11.0%) were born outside Canada (Table 1). There was a greater proportion of boys in the intervention group than in the control group (265 [51.5%] vs 228 [45.6%]). Teaching experience was slightly higher in the intervention group (mean [SD], 17.01 [8.54] vs 13.82 [7.89] years), and more students in the intervention group had teachers born outside Canada (124 [24.1%] vs 82 [16.4%]). About half of all students in both groups had teachers with no prior experience teaching outdoors (intervention, 219 [42.5%]; control, 238 [47.6%]). The socioeconomic disadvantage indicator of the schools was about 1 point higher in the control group than in the intervention group (mean [SD], 5.81 [2.56] vs 4.18 [3.32]) (Table 1). At immediate postintervention follow-up (T1), attrition in both groups was minimal (Figure 1).

**Table 1.** Participant and School Characteristics at Baseline

Characteristic	Students^a^	
	Intervention group (n = 515)	Control group (n = 500)
**Student characteristics**		
Sex^b^		
Female	248 (48.2)	259 (51.8)
Male	265 (51.5)	228 (45.6)
Birthplace		
Canada	474 (92.0)	413 (82.6)
Outside Canada	36 (7.0)	76 (15.2)
Primary language		
English and/or French	471 (91.5)	402 (80.4)
Neither English nor French	43 (8.3)	90 (18.0)
Disability or special needs status^c^		
Yes	121 (23.5)	122 (24.4)
No	383 (74.4)	371 (74.2)
Age, mean (SD), y^d^	10.9 (0.73)	11.0 (0.76)
**Teacher characteristics**		
Sex		
Female	406 (78.8)	391 (78.2)
Male	109 (21.2)	109 (21.8)
Birthplace		
Canada	391 (75.9)	418 (83.6)
Outside Canada	124 (24.1)	82 (16.4)
Teaching experience, mean (SD), y	17.01 (8.54)	13.82 (7.89)
Outdoor teaching experience in past 3 y		
No	219 (42.5)	238 (47.6)
Yes	296 (57.5)	262 (52.4)
**School characteristics**		
Socioeconomic disadvantage indicator, mean (SD)^e^	4.18 (3.32)	5.81 (2.56)
Green space as captured by NDVI, mean (SD)^f^	0.46 (0.08)	0.44 (0.08)

Abbreviation: NDVI, Normalized Difference Vegetation Index.

^a^ Data are presented as number (percentage) of students unless otherwise indicated.

^b^ Self-reported. Missing student responses were imputed with teacher reports of student sex.

^c^ Teachers reported whether the student was in 1 of 3 categories established by the Quebec Ministry of Education: (1) vulnerability factors likely to influence learning or behavior and possibly at risk without rapid intervention, even if no official diagnostic code; (2) disabilities, social maladjustments, or learning difficulties with an official diagnostic code; and (3) social maladjustments or learning difficulties without a diagnostic code who benefit from an intervention plan.

^d^ Age on March 6, 2023.

^e^ Calculated by Quebec as the proportion of mothers without higher education and of parents who are unemployed, by school postal code.

^f^ Quantifies the density of green vegetation associated with the school’s postal code. Scores range from −1 to 1, with higher values indicating greater density of green vegetation. Information was sourced from the Canadian Urban Environmental Health Research Consortium,^28^ reported within a 250-m buffer zone.

### Implementation Adherence

Teacher logbooks (eTable 1 in Supplement 2) indicated a mean (SD) of 116 (33.8) minutes (range, 65-217 minutes) per week of teaching outdoors, mostly in a schoolyard, park, or wooded area. Only 192 of 471 students in the intervention group (40.8%) received the full 2-hour weekly requirement. Teachers completed a mean (SD) of 7.5 (3.24) mental health activities (range, 0-15 activities); 6 of 26 teachers (23.1%) met the required 10 mental health activities, and 12 of 26 (46.2%) achieved 80% adherence (≥8 activities). For academic subjects, teachers conducted a mean (SD) of 5 (3.6) tool kit activities (range, 0-13 activities) and 11 (5.5) teacher-initiated activities (range, 2-21 activities). In the control group, 13 of 27 teachers (48.1%) reported outdoor activities at least once, with 5 classes (77 students) engaging in over 2 hours per week (eMethods 4 in Supplement 2).

### Outcomes

Primary outcome results (ie, change in mental health immediately after the intervention [T1]) were not statistically significant (Table 2). The adjusted mean difference in SBQ scores between the intervention and control groups for externalizing symptoms was −0.04 (95% CI, −0.13 to 0.04) in the intent-to-treat analysis and −0.06 (95% CI, −0.16 to 0.04) in the per-protocol analysis. Other mental health results were similar to the results for externalizing symptoms (Table 2).

**Table 2.** Changes in Student Mental Health, as Measured by the Social Behavior Questionnaire and Adjusted Using Clustered Mixed Modeling^a^

Symptom	Observed score, mean (SD)				Adjusted change, mean (95% CI)^b^			
	Intervention group		Control group		Intent to treat^c^	*P* value^d^	Per protocol^e^	*P* value^d^
	Baseline	Follow-up	Baseline	Follow-up				
**Teacher reports**								
Internalizing	0.60 (0.44)	0.48 (0.37)	0.62 (0.41)	0.55 (0.39)	−0.06 (−0.13 to 0.01)	.11	−0.07 (−0.17 to 0.04)	.20
Externalizing	0.35 (0.41)	0.31 (0.38)	0.33 (0.38)	0.32 (0.37)	−0.04 (−0.13 to 0.04)	.30	−0.06 (−0.16 to 0.04)	.23
Social problems	0.73 (0.40)	0.75 (0.38)	0.79 (0.36)	0.77 (0.35)	−0.04 (−0.14 to 0.07)	.48	−0.05 (−0.14 to 0.04)	.25
**Student reports**								
Internalizing	0.62 (0.37)	0.58 (0.37)	0.64 (0.36)	0.60 (0.37)	0.00 (−0.05 to 0.05)	.98	−0.02 (−0.08 to 0.04)	.42
Externalizing	0.45 (0.32)	0.42 (0.30)	0.45 (0.31)	0.44 (0.31)	−0.02 (−0.05 to 0.02)	.41	−0.02 (−0.06 to 0.03)	.45
Social problems	0.44 (0.36)	0.44 (0.37)	0.48 (0.37)	0.46 (0.38)	0.02 (−0.03 to 0.06)	.49	0.01 (−0.03 to 0.06)	.56

^a^ The Social Behavior Questionnaire evaluates frequency of symptoms over the past 2 months on a 3-point scale (never or not true = 0, sometimes or somewhat true = 1, and often or very true = 2). A continuous score was retained for each subscale.

^b^ Adjusted for student sex, age, birth outside Canada, and primary language not English or French; teacher born outside Canada and teacher’s years of teaching experience; and school socioeconomic disadvantage indicator and Normalized Difference Vegetation Index (quantified by school postal code within a 250-m buffer zone).

^c^ Teacher-reported internalizing symptoms, n = 928; teacher-reported externalizing symptoms, n = 928; teacher-reported social problems, n = 918; student-reported internalizing symptoms, n = 940; student-reported externalizing symptoms, n = 941; and student-reported social problems, n = 930.

^d^ Significance for mixed-model analyses was set to *P* = .025 (2/.05) to adjust for co–primary outcomes.

^e^ Classes in the intervention group that did not engage in 80% of the intervention time were excluded. Teacher-reported internalizing symptoms, n = 746; teacher-reported externalizing symptoms, n = 746; teacher-reported social problems, n = 746; student-reported internalizing symptoms, n = 785; student-reported externalizing symptoms, n = 786; and student-reported social problems, n = 786.

Due to high attrition (Figure 1), we were unable to analyze the 3-month follow-up data (T2). We therefore used clustered mixed-model analysis (Table 2), which is better for 2 time points than the originally planned longitudinal analysis of covariance. Secondary outcomes in both intent-to-treat and per-protocol analyses showed no statistically significant effects for depressive symptoms, positive affect, negative affect, nature connectedness, or environmental efforts and attitudes (Table 3).

**Table 3.** Changes in Secondary Outcomes, Adjusted Using Clustered Mixed Modeling^a^

Student-reported outcome	Observed score, mean (SD)				Adjusted change, mean (95% CI)^b^			
	Intervention		Control					
	Baseline	Follow-up	Baseline	Follow-up	Intent to treat^c^	*P* value^d^	Per protocol^e^	*P* value^d^
Depressive symptoms	18.29 (4.34)	18.19 (4.62)	18.43 (4.35)	18.29 (4.69)	0.13 (−0.50 to 0.77)	.66	−0.06 (−0.72 to 0.60)	.83
Positive affect	33.14 (6.83)	32.70 (6.98)	33.07 (7.02)	33.13 (7.66)	−0.43 (−1.44 to 0.57)	.38	−0.31 (−1.43 to 0.81)	.57
Negative affect	20.19 (6.83)	20.44 (7.46)	20.00 (6.78)	21.15 (7.98)	−0.70 (−1.74 to 0.33)	.18	−0.99 (−2.16 to 0.17)	.09
Nature connectedness	5.69 (1.12)	5.51 (1.25)	5.57 (1.19)	5.33 (1.34)	0.09 (−0.08 to 0.26)	.28	0.14 (−0.07 to 0.35)	.17
Environmental efforts	3.72 (0.77)	3.67 (0.79)	3.64 (0.81)	3.58 (0.84)	0.01 (−0.13 to 0.14)	.90	0.01 (−0.13 to 0.16)	.86
Environmental attitudes	3.22 (0.59)	3.03 (0.66)	3.09 (0.65)	2.97 (0.73)	−0.04 (−0.13 to 0.05)	.37	−0.04 (−0.14 to 0.07)	.45

^a^ Secondary outcomes were changes in mental health and relationship to the environment, as self-reported by students at baseline and at 12 weeks and 3 months after the intervention. Measures over the past 2 weeks included positive and negative affect (20-item Positive and Negative Affect Schedule for Children, on a 5-point Likert scale; very slightly or not at all = 1 and extremely = 5)^24^ and depressive symptoms (13-item Children’s Depression Inventory–Short Version, on a 3-point scale: I hate myself = 1, I don’t like myself = 2, and I like myself = 3).^25^ Environmental outcomes included nature connectedness (6-item Nature Connection Index)^26^ and proenvironmental behaviors (eg, recycling) and efforts (eg, volunteering), as measured by 6 items from a previous study.^27^

^b^ Adjusted for student’s sex, age, born outside Canada, and primary language not English or French; teacher born outside Canada and years of experience as a teacher; and school socioeconomic disadvantage indicator and Normalized Difference Vegetation Index (quantified by school postal code within a 250-m buffer zone).

^c^ Depressive symptoms, n = 937; positive affect, n = 935; negative affect, n = 931; nature connectedness, n = 940; environmental efforts, n = 940; and environmental attitudes, n = 940.

^d^ Significance for mixed-model analyses for secondary outcomes was set at *P* = .05.

^e^ Classes in the intervention group that did not engage in 80% of the intervention time were excluded. Depressive symptoms, n = 783; positive affect, n = 781; negative affect, n = 778; nature connectedness, n = 785; environmental efforts, n = 786; and environmental attitudes, n = 785.

### Moderation Analyses

We explored the potential moderating effects of sex, disability or special needs status, green space, school socioeconomic disadvantage indicator, and prior outdoor teaching experience (eTable 2 in Supplement 2). With the exception of sex, we did not find statistically significant effects (1 of 30 models [3.3%]).

### Post Hoc Analyses

**Figure 2.**
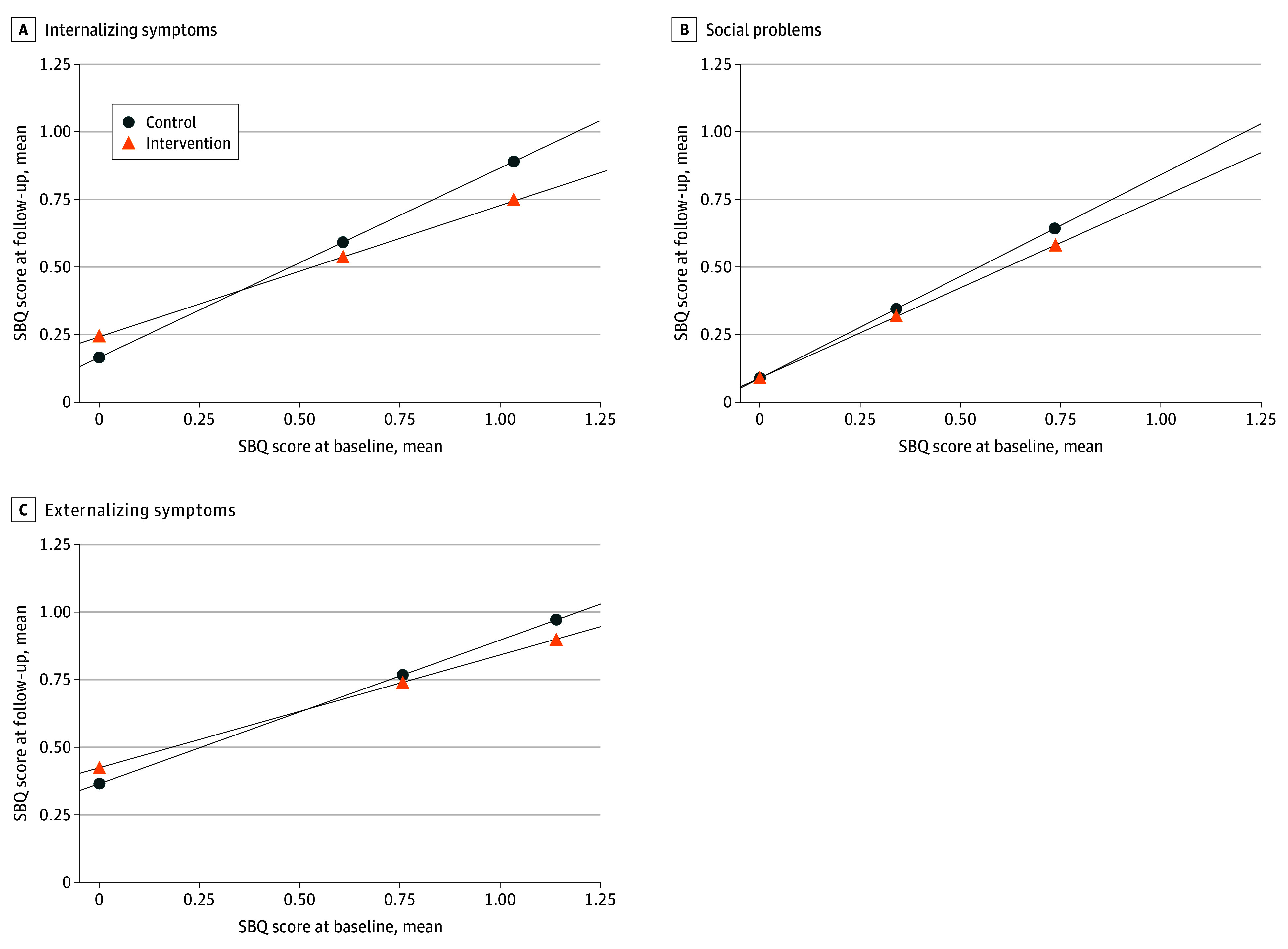
Mental Health Symptoms as Reported by Teachers at Postintervention Assessment as a Function of Mental Health Symptoms at Baseline in the Intervention and Control Groups Symptoms were assessed using the Social Behavior Questionnaire (SBQ). The SBQ evaluates frequency of symptoms over the past 2 months on a 3-point scale (never or not true = 0, sometimes or somewhat true = 1, often or very true = 2). We retained a continuous score for each subscale. Results were adjusted for student sex, age, birth outside Canada, and primary language other than English or French; teacher born outside Canada and teacher’s years of teaching experience; and school socioeconomic disadvantage indicator and Normalized Difference Vegetation Index (quantified by school postal code within a 250-m buffer zone).

Post hoc analyses revealed low levels of mental health symptoms at baseline, with low variability. We explored effectiveness as a function of baseline symptoms. We did not find significant interactions for student self-reported measures. However, for teacher-reported measures, the intervention was effective for students with higher levels of symptoms at baseline (all *P* < .05 for interaction). For example, for children with baseline scores 1 SD above the mean, internalizing symptoms decreased by 0.38 SD (mean change, −0.15; 95% CI, −0.23 to −0.06; *P* < .001), externalizing symptoms by 0.21 SD (mean change, −0.08; 95% CI, −0.17 to 0.01; *P* = .07), and social problems by 0.19 SD (mean change, −0.07; 95% CI, −0.18 to 0.04; *P* = .21) in the intervention group vs control group (Figure 2).

## Discussion

In what was, to our knowledge, the first cluster randomized clinical trial to test the effectiveness of a 12-week, nature-based outdoor program for improving child mental health in grades 5 and 6 in 33 elementary schools (1015 students) throughout Quebec, Canada, we found no overall changes in children’s mental health immediately after the intervention (ie, at 12 weeks) in the intent-to-treat or per-protocol analysis. Post hoc analyses suggested a small favorable outcome for teacher-reported internalizing symptoms, externalizing symptoms, and social problems for children with higher symptom levels at baseline. Notably, the intervention did not worsen mental health. Together, these findings indicate that the intervention may, at a minimum, be helpful in reducing disparities in mental health in children with preexisting symptoms. Teachers in both groups expressed interest in teaching outdoors in natural surroundings.

Post hoc moderation analyses revealed low levels of mental health symptoms at baseline. As such, we realized that the intervention could not decrease problems that were already low or nonexistent. Our findings are timely given advocated nature outings to improve well-being.^31^ Outings in nature, preferably 2 hours per session,^32^ were 1 of the top 5 online suggestions to improve well-being according to a meta-analysis.^31^ Only 3 adequately powered randomized experiments in nonclinical populations supported this claim, and none were in a school setting.^31^ Furthermore, previous studies were limited by their within-participant design, small sample size (N<78) with few child participants, and lack of preregistration.^33,34,35^ We used a large sample size clustered at the school level in a daily-life elementary school setting. Absence of statistically significant differences for all children suggests that further program modifications, adjustments, and research would be beneficial before formally recommending the program for universal implementation.

However, our results showed small benefits in select subgroups (children with prior mental health symptoms). This aligns with nature-centered therapeutic approaches designed for individuals experiencing difficulties^3,36,37,38^ and initiatives, such as “nature prescriptions,” by health care professionals.^7^ Our results corroborate a seminal study showing that a walk in the park vs on city streets alleviated symptoms in children with attention-deficit/hyperactivity disorder, although that study had effect sizes comparable to those for medication.^14^ A follow-up randomized clinical trial (N = 24) found no medication-equivalent effects.^39^ Though we did not specifically measure attention during or immediately after exposure, teachers in the intervention group found that after a short-term exploration period, student concentration improved dramatically. Our findings regarding children with mental health symptoms are also consistent with studies indicating that school-based nature interventions benefited participants with specific characteristics (eg, boys)^12^ or those from disadvantaged neighborhoods.^11^ These findings highlight nature as important in alleviating existing internalizing symptoms, such as anxiety and depression.^37,40^

The American Academy of Pediatrics and the Canadian Paediatric Society advocate for equity in mental health and nature exposure.^5,6^ Although our study did not demonstrate benefits for children without preexisting mental health symptoms, there may be unmeasured advantages. First, the intervention may be preventive, potentially mitigating future challenges.^41^ Notably, many schools in disadvantaged areas have less green space nearby,^42^ and our study was underpowered to look at disadvantaged areas specifically. Second, there may be short-term improvements in mood for all children^33,34,35,43^ that the 8-week window of the SBQ would not reflect. Third, the intervention may indirectly lead to lifestyle modification, such as reduction in sedentary behavior (eg, walking to outdoor location, love of nature), which may improve outcomes such as fitness, academic motivation,^44^ self-regulation,^45^ or obesity. Further research on benefits is warranted.

Of note, the intervention group faced challenges meeting the benchmark of 2 hours per week outdoors, and only 46.2% of teachers reached 80% adherence to the 10 requested mental health activities. Teachers cited adverse weather conditions and lack of time in the school curriculum as barriers to implementation. Also, encouragement of teacher-initiated activities, while promoting flexibility and teacher autonomy, introduced additional heterogeneity in the fidelity of the intervention. In the control group, 48.1% of teachers engaged in teaching outdoors, sometimes with frequencies comparable to the intervention group. Heterogeneity in implementation may have reduced group differences.

During and after the intervention, we received emails from control group teachers motivated to teach outdoors. Likewise, teachers in the intervention group expressed enjoyment in outdoor teaching. The fact that no classes were lost at follow-up (all students received the intervention) supports the acceptability and attractiveness of the intervention.

### Strengths and Limitations

This study has strengths. Our robust design marks this study, to our knowledge, as the inaugural cluster randomized clinical trial to test the effectiveness of a nature-based outdoor intervention for reducing mental health symptoms in individuals aged 10 to 12 years in daily-life settings. As such, it fills a gap in the literature. We used a large sample size (perhaps the largest to date), registered the study, and published a peer-reviewed protocol.^18^ The large sample size in French-language schools throughout Quebec suggests generalizability in that population. We used a multi-informant approach to assessing child mental health, incorporating diverse perspectives to provide a comprehensive understanding of the outcomes. Moreover, the activity tool kit was positively appraised by teachers. The tool kit enriches the intervention and can be used elsewhere in educational settings, particularly the mental health components, such as mindfulness, which are not usually taught in elementary school. Furthermore, we included schools of varied socioeconomic levels, as reflected by the mean socioeconomic disadvantage indicator between 4 and 5 on a range of 0 to 10.

Our study also has several limitations. Blinding was not feasible due to the nature of the intervention, implying putative social desirability bias in reporting of outcomes. Second, we relied on teacher logs to measure implementation, which is less precise than more objective measures (eg, video recordings). Third, we had no information on the quality of the chosen green space. Fourth, immediate postintervention assessment (at 12 weeks) of improvements over the past 8 weeks meant a measure of partial exposure to the intervention (first 4 weeks), obscuring longer-term cumulative improvement over time. Fifth, as the intervention featured mental health activities in addition to natural settings, potential benefits could be attributed to either or both. As adherence to mental health activities was low, possible benefits were likely attributable to nature. Furthermore, many teachers in the control group engaged in outdoor teaching. Sixth, participants did not constitute a population-based representative sample. Moderation analyses, however, showed that results generally did not vary by green space and socioeconomic status. Seventh and importantly, the 3-month follow-up coincided with a return to school from summer holidays, when students were in different classes, explaining attrition rates at T2. For longer-term assessment, studies should be scheduled earlier in the school year.

## Conclusions

In this cluster randomized clinical trial, we found that 12 weekly 2-hour school sessions in green space did not reduce mental health symptoms in students aged 10 to 12 years in either the per-protocol or the intent-to-treat analysis. Of note, symptom levels at baseline were already low, as the intervention was implemented in a daily-life elementary school setting in grades 5 and 6. Post hoc moderation analyses suggested small benefits to children with higher levels of internalizing symptoms, externalizing symptoms, or social problems at baseline. Furthermore, the intervention was low cost and caused no harm. Participants reacted favorably. As parks and green spaces are freely enjoyable and ubiquitous, implementation of nature-based interventions, such as the Open Sky School Program, can be encouraged by policymakers, educators, and stakeholders. However, future studies are warranted to better understand the potential effectiveness of these interventions as therapeutic or preventive measures, especially among children with mental health symptoms.

## Notes

Supplement 1.
Trial Protocol

Supplement 2. **eMethods 1.** Deviation From Original Protocol **eMethods 2.** Description of Measures **eMethods 3.** Description of the Intervention **eMethods 4.** Contamination Effects in the Intervention and Control Groups **eTable 1.** Characteristics of the Intervention Group—Teacher Logbook **eTable 2.** *P* Values for the Interactions Between Moderators and Intervention Effectiveness
eReferences

Supplement 3.
Data Sharing Statement
