# Supplementary material for: A Nature-Based Intervention and Mental Health of Schoolchildren: A Cluster Randomized Clinical Trial
Source: JAMA Netw Open. 2024 Nov 15;7(11):e2444824. doi: 10.1001/jamanetworkopen.2024.44824 (PMC11568460; doi:10.1001/jamanetworkopen.2024.44824)
Supplement: Supplement 1. — Trial Protocol [file jamanetwopen-e2444824-s001.pdf]

Effectiveness of the Open Sky School Program, a nature-based intervention, on children's mental health: A cluster randomized controlled trial

Principal investigator: Marie-Claude Geoffroy
Co-principal investigators : Sylvana Côté, Catherine Malboeuf-Hurtubise, Jean-Philippe Ayotte-Beaudet,
Investigators : Tianna Loose, Nicholas Chadi, Lise Gauvin, Geneviève Lessard, Isabelle Ouellet-Morin.

Partners:
Mères au front
Espace pour la Vie
Observatoire pour l'Éducation et la Santé des enfants (OPES)

Funding Agency: Canadian Institutes of Health Research (CIHR)
Competition: Operating Grant: Understanding and mitigating the impacts of the COVID-19 pandemic on children, youth, and families in Canada - $149,558

Funding Agency: Fonds de Recherche du Québec – Société et Culture (FRQ-SC) via the Observatoire pour l'Éducation et la Santé des enfants (OPES) - $150,000

Funding Agency: Manulife
Competition: Global Community Investment - $150,000

This IRB-approved protocol is a free translation and adaptation from French to English, carried out by the authors of the manuscript. The original French protocol was partially translated and published as an open-access, peer-reviewed article in *BMC Public Health*.[1] As a result, there is some overlap between the Methods section of the text below and the Methods sections of protocol previously published. This procedure and disclosure was conducted in accordance with the best practices outlined by the Text Recycling Research Project.[2]

The protocol was initially approved on April 28, 2022.

Lay abstract

How can we reduce the impact of the COVID-19 pandemic on children's mental health? We propose the *Open Sky School*, a nature-based intervention applied in the school setting. Currently, there is increasing enthusiasm for outdoor education in schools, especially since the onset of the COVID-19 pandemic. Could such nature-based interventions have salutogenic effects? Experimental studies conducted worldwide show that contact with nature (e.g., urban park or forest) improves mental health. Preliminary results suggest that this is also the case for children when they come into contact with nature during school hours. However, no experimental studies have tested the benefits of spending time in nature at school as a strategy for improving children's mental health.

To fill the gaps in the existing literature, we will conduct a clustered randomized controlled trial to test the effectiveness of the *Open Sky School* program. The *Open Sky School* is a nature-based intervention applied in the school setting to promote students' and teachers' mental health. The intervention will span 12 weeks, two hours per week. We will recruit 80 to 100 5th and 6th grade elementary school classes from schools in Quebec. The intervention will include mental health promotion activities in and through nature (e.g., cooperation, compassion/empathy, mindfulness) and nature-based educational activities. We will evaluate the effectiveness of this intervention on children's mental health. The results could help decision makers implement education programs involving contact with nature to promote mental health. Our results could provide empirical evidence of such practices that come at little cost and have a high potential for deployment at the population level.

Research Ethics Board Approved Protocol for the Open Sky School: Effectiveness of a nature based intervention in the school setting on improving mental health and healthy habits among children

1. Overview and impact

Good mental health during childhood paves the way for good physical and mental health throughout life.[3] Before the pandemic, 1 in 3 children experienced emotional and/or internalized and/or externalized behavioral problems, such as anxiety, depression, inattention/hyperactivity, and opposition.[4] However, since the COVID-19 pandemic, the number of emergency room visits and prescriptions for medication for mental health problems in young people has increased significantly in Quebec compared to previous years,[5] and particularly among children from disadvantaged backgrounds.[6] In addition to school closures and distance learning, many children experienced the impact of parental stress, as well as a marked decrease in social contact with friends and family.[7] To mitigate the pandemic's negative impact on children's mental health, it is crucial to identify and implement feasible interventions that effectively improve mental wellbeing.

Scientific studies with experimental and quasi-experimental designs conducted around the world, including by our team, show that contact with nature (e.g., walking in a nearby park versus in the city) improves adults' mental health,[8,9] by reducing depression, anxiety, suicidal ideation and inattention. As such, a growing number of initiatives have emerged encouraging individuals to spend time in nature to improve their mental health, including during the COVID-19 pandemic.[10] This enthusiasm is echoed in the education sector, where a growing—though still limited—number of teachers are incorporating outdoor education with aims similar to nature-based interventions used in therapeutic contexts. However, the impact of time spent in nature on children's mental health remains inadequately understood, largely due to a scarcity of experimental studies.

This research project will help fill this gap by testing the benefits of time spent in nature, in the school setting, on mental health symptoms of 5th and 6th grade elementary school students in Quebec. We will conduct a clustered randomized controlled trial to test the effectiveness of a 12-week intervention, called the *Open Sky School*, which will be implemented by teachers. The *Open Sky School* consists of 24 hours of time spent in nature (i.e., nearby park or other natural green environment such as a waterfront or forest) during school hours. The students will be exposed to nature two hours per week for a period of 12 weeks, from March to June 2023. The waitlist control group will be composed of students in classes continuing usual teaching practices from March to June 2023. The control group will have access to the intervention, including 10 mental health activities accessible on a website, in the fall of 2023. Teachers in the experimental group will have access to training and coaching during the intervention period to facilitate the integration of mental health and pedagogical activities into the regular school curriculum. This will help facilitate teachers' implementation of the intervention.

Outdoor education involving contact with nature is rapidly gaining interest in school settings, but the actual potential of these initiatives to promote young people's mental health is poorly understood. Therefore, it is vital to generate evidence to guide decisions about which types of mental health promotion interventions we should prioritize in schools, as well as the appropriateness/relevance of such initiatives. In addition, given the current post-pandemic context and the increased risk of infection in children under 12, time spent in nature outside at school is a promising strategy to reduce the risk of viral exposure and thus help keep schools open during the 2022-2023 academic year, while reducing the risk of spreading COVID-19 and other infections in children. Our results will help provide decision-makers with the evidence needed to decide which outdoor education programs to implement in a post-pandemic context. The evidence generated can then be mobilized on a wider scale, across the province and even the country.

2. Current state of knowledge: justifying nature-based intervention in schools to improve children's mental health

Our research team is primarily interested in the benefits of time spent in nature on children's mental health, at the population level. However, we are also concerned with implementing an intervention that can realistically and seamlessly be integrated within the school setting. This will enhance the relevance of the intervention. Additionally we will focus on integrating pedagogical activities to better fit teachers' needs. Thus, by combining these two approaches, we can ensure that we will promote better mental health while implementing an intervention that enables teachers to comply with the cycle 3 (5th and 6th grade) Quebec school curriculum. The following is a summary of the existing literature on 1) the therapeutic and salutogenic impacts of contact with nature on mental health, and 2) outdoor education in primary schools.

2.1. What do we know about the effects of contact with nature on mental health?

First, observational studies examining the associations between exposure to nature (measured by the vegetation index surrounding the place of residence) during childhood and mental health in adulthood report associations between greater presence of green vegetation density and lower levels of depression, anxiety and suicide.[11] In children, some observational studies show that a greater amount of vegetation in the neighbourhood is associated with fewer depressive symptoms, anxiety,[12] and inattention/hyperactivity,[13] while also providing more opportunities for physical activity.[14] Our own preliminary results on a birth cohort of children born in Quebec in 97/98 demonstrated that children living in an urban neighbourhood with a high vegetation index had fewer symptoms of inattention and hyperactivity/impulsivity during adolescence than those who grew up in a less green neighbourhood,[15] and that these associations held after controlling for household and neighborhood socioeconomic status. Second, intervention studies carried out among adults reveal benefits of contact with nature in reducing symptoms of depression and anxiety.[9] For instance, a systematic review of 12 interventional studies based on quasi-experimental design showed that walking in natural environments (e.g., parks) reduced anxiety symptoms in adults more than walking in urban city settings.[16] In addition, a meta-analysis of 16 studies evaluating the effects of an intervention based on spending time in the forest showed reductions in anxiety symptoms.[17] In all, there is evidence of benefits of spending time in nature on mental health, whether the natural space is a forest or an urban park.[18]

However, few intervention studies, most of which are quasi-experimental, have documented the effects of a nature exposure among children. Furthermore, such studies carried out among children targeted those with pre-existing mental health problems.[19] For example, a double-blind, controlled, within-subject study was carried out among children aged 7 to 12 years with a diagnosis of ADHD. The study suggested that 20-minute walks in urban parks reduced concentration problems among participants more than walks in urban environments, such as streets in their neighborhood.[20] Notably, the effect sizes observed were large (0.52 to 0.77) and comparable to the effects of medication. Despite these encouraging results, these findings are emerging and limited. In addition, there is a lack of strong evidence to demonstrate the beneficial impact of contact with nature on children's mental health. This project therefore aims to generate knowledge to document the effects of exposure to nature on the mental health of children attending schools in Quebec in the aftermath of the COVID-19 pandemic.

2.2. Explanatory mechanisms

Although knowledge does not allow us to identify the bio-psycho-social mechanisms involved in the relationship between contact with nature and mental health, some explanations have been put forward: (1) natural spaces can reduce stress (e.g., cortisol) and increase attentional capacities, self-control and problem-solving skills, which in turn promote a restoration of psychological wellbeing;[9]

(2) contact with nature can contribute to improving psychosocial adaptation and mental health by means of increasing physical activity and social connections;[21,22] and (3) natural spaces have lower exposure to air and noise pollution which is associated with mental health benefits in the short and long term.[23,24]

2.3. What do we know about the effects of outdoor education on children's mental health?

Traditionally, studies examining the impact of outdoor education have primarily focused on educational attainment in various subjects rather than on youth mental health.[25] While positive effects have been observed on physical activity,[26] social skills, and self-esteem,[25] the benefits to mental health remain largely unexplored. It should be noted that outdoor education includes any form of teaching and learning that takes place outside the walls of the school, regardless of the presence of natural spaces. Nevertheless, despite these limitations and the lack of empirical evidence generated from randomized controlled trials, there are a few quasi-experimental studies that have documented the effects of outdoor education on children's mental health.[27,28] For example, a first study conducted in Sweden carried out among children aged 6 to 12 years used a non-equivalent quasi-experimental design to test if an outdoor education program (e.g., schoolyard) intervention (n=121) reduced mental health symptoms compared to teaching as usual (n=109). The intervention was carried out daily, 1 hour per day for 6 months during which students learned the classic core subjects outdoors by manipulating elements of nature (e.g., branches and stones used to represent mathematical concepts as opposed to textbook content). Findings suggested that the intervention significantly reduced internalizing and externalizing symptoms, but only among boys.[28] Subsequently, a second quasi-experimental study aimed to assess the potential benefits of outdoor education on the mental health of children aged 9 to 11 (n=631) in comparison to teaching as usual. Results suggested that the intervention increased prosocial behaviors among all children and reduced hyperactivity and peer problems, but only among children from underprivileged backgrounds.[27]

2.4. Added value of our study

In light of the available results and the previously identified gaps, we propose to implement a clustered randomized controlled trial of an intervention combining both the component of contact with natural spaces and outdoor education. We will evaluate the impact of the intervention on the mental health of 5th and 6th grade elementary students.

3. Study objectives

The primary objective is to test the benefits of time spent in nature on the mental health of 5th and 6th grade elementary students. To be eligible, students must be aged 10-12 years and attend a school with access to a natural space (i.e., park) within a 1 kilometer radius. We hypothesize that students exposed to our 12-week intervention in the 2022-2023 school year will have fewer internalizing (e.g., depression, anxiety) and externalizing (e.g., opposition, inattention/hyperactivity/impulsivity) symptoms and social problems (e.g., bullying/victimization) compared to children in the waitlist control group, who will follow teaching practices as usual. The ***secondary objectives*** are to study the intervention's effectiveness on children's nature connectedness and other indicators of students' and teachers' wellbeing. Finally, the exploratory objectives of the project are to assess if the effects of the intervention are maintained in the medium-term (i.e., 3 months later) and to examine potential moderating effects, according to various characteristics of (a) the child, i.e., sex, the presence or absence of a diagnosed mental health disorder in the student's file; and (b) the school, e.g., the geographical area, and the vegetation index of greenness surrounding the school.

4. Methods

4.1. Design

We will implement a clustered randomized controlled trial with a parallel repeated-measures design. In the trial, 80 to 100 schools with 5th and 6th grade classes will be recruited across Quebec and be randomly assigned to one of the following conditions: 1) *Open Sky School* intervention (intervention group) and 2) teaching as usual (waitlist control group, who will receive the intervention the following school year if it is effective). We will randomize at the school level rather than at the class level to allow for more than one class within the same school to take part in the project while avoiding possible contamination effects. In the context of our project we define our nature intervention by exposing children to natural environments during school hours and including any activity that promotes learning or mental health. The available natural space must be located near the school, within a 1 kilometer radius, and can be, for instance, an urban park, wooded area or waterfront. In other words, we are interested in engaging children in nature (in urban or rural areas) and providing access to academic or mental health activities in order to promote their wellbeing.

4.2. Randomization

Randomization will occur after baseline assessments. Once randomization has occurred, it will not be possible to blind researchers and participants within our design. A simple computer algorithm will be used to randomly allocate schools to two parallel and balanced conditions (intervention or control; 1:1 while considering class sizes) by an independent researcher not involved in the study. This will ensure that all schools have an equal opportunity to be allocated into one of the two groups. In the event that multiple teachers from the same school consent to participate, they will be allocated to the same experimental condition to avoid contamination effects.

Figure 1. Flow chart of inclusion of participants.

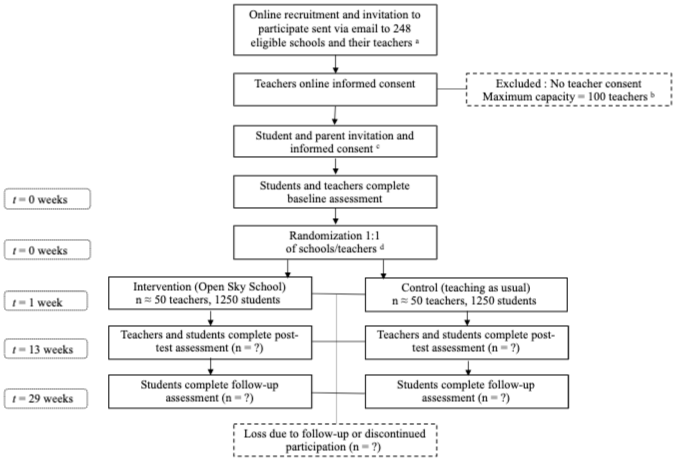

Note. [a] 280 schools in 29 school boards were initially eligible. 4 school boards encompassing 32 schools did not agree to participate and were therefore removed from the recruitment process. [b] If more than 100 teachers consent to participate, we will randomly select 100 teachers to participate in the trial. [c] Parents and students must provide informed consent for students to participate in the assessments. Students who do not participate in assessments will still participate in the Open Sky School program. [d] In the event that multiple teachers from the same school consent to participate, they will be allocated to the same experimental condition to avoid contamination effects. If recruitment targets at the pre-selected school are not met, we will advertise online.

4.3. Measurement times

Children's mental health will be assessed by the teacher and self-reported by the child first before randomization (T1: March 2023), then after the intervention at the end of the school year (T2: June 2023) during class time. Since we anticipate the intervention's effects will be maintained in the medium term, children will fill out questionnaires again (self-reported mental health) (T3: September 2023), i.e. 3 months after the intervention ends. For T3, we will contact parents so that the children can fill in the questionnaires online. The clustered randomized controlled trial will be conducted without blinding participants to conditions, as it is not possible to implement a double-blind design with teachers and children.

4.4. Recruitment strategy and timeline

Participating schools will be recruited from a list of 280 French language elementary schools in the Canadian province of Quebec, enrolled in a larger study conducted by the Observatory for Children's Education and Health, which aims to document the consequences of the COVID-19 pandemic on 4th grade children's academic achievement (www.observatoireenfants.ca/en). Eligible schools will receive an initial invitation letter by email (December 2022), containing a 90 second informative video, which they will be asked to distribute to their 5-6th grade teachers. In the invitation letter, 5-6th grade teachers will be invited to attend an online information session describing their involvement in the *Open Sky School* project. All interested teachers will be sent an information and consent form. Once teachers consent to take part in the study, they will distribute information and consent forms to students and students' parents. To maximize recruitment potential, we will also post a recruitment flyer via social networks inviting interested participants to contact us via email to receive the aforementioned information describing their involvement in the project.

Baseline assessment will take place from March 6 to 10, 2023 and post-test assessments will be conducted from June 5 to 12, 2023. The weekly 2-hour nature visits will take place from March 13, 2023 to June 2, 2023. The follow-up assessments will be administered from September 18 to 23, 2023. To encourage participation throughout the study, teachers will be compensated $100 CAD for evaluating their students' mental health symptoms before and after the intervention (up to $200 CAD total). Children can choose to participate in a draw to win gift cards to a local bookstore. More specifically, we will draw two $50 CAD gift cards per class for children who complete questionnaires at baseline and immediate follow up, and ten $100 CAD gift cards for children who complete the follow-up assessment. Figure 1 shows a flow chart of the inclusion of participants.

4.5. Data collection, analysis and dissemination

Data will be collected online using Qualtrics, a widely used software in analytics and research. Confidential data (e.g., name, zip code) will be uploaded to a private and secure computer and then periodically erased from the server. The data file to be analyzed will contain only the anonymous (numeric) identifier and the questionnaire reponses. The file linking the identifiers to the data file will be kept on an encrypted USB key, protected by a password and kept in a locked safe. The safe will be kept in Dr. Geoffroy's and research coordinator's work office. Only Dr. Geoffroy and the coordinator will have access to it. When data collection is complete, the data will be downloaded to a private computer and then deleted from Qualtrics. Members of the research team responsible for analyzing data will only have access to this anonymous dataset which will be used solely for the purpose of establishing and disseminating research findings. Results will be disseminated to researchers, stake-holders, policy-makers and participants via scientific publications, oral presentations, interviews, and

outreach. All communications related to the project will be made available publicly on the official website https://www.ecolecielouvert.ca/.

5. Instruments for measuring primary and secondary outcome measures

Measures	Measure	Participant	T1	T2	T3
**Sociodemographics**		Student	•		•
**For children**					
Mental health					
**General mental health**	Social Behaviour Questionnaire	Student	•	•	•
**General mental health**	Social Behaviour Questionnaire	Teacher	•	•	•
**Depressive symptoms**	Children’s Depression Inventory-Short Version	Student	•	•	•
**Positive and negative affect**	Positive and Negative Affect Schedule-Child	Student	•	•	•
Relationship with the environment					
**Connection with nature**	Nature Connection Index (NCI)	Student	•	•	•
**Pro-environmental behaviours**	Taken from Keith et al.	Student	•	•	•
Program evaluation					
**Appreciation of the program**	Ad hoc	Student		•	
**For teachers**					
**Wellbeing**	World Health Organization Wellbeing Index (WHO-5)	Teacher	•	•	
**Teachers' enjoyment of teaching outdoors**	Enjoyment of Teaching Mathematics Scale - adapted	Teacher		•	
**Positive and negative affect**	Positive and Negative Affect Schedule	Teacher	•	•	
**Moderators**					
**Qualifications and experience of teachers**	Ad hoc	Teacher	•		
**Teachers' prior experience with outdoor education**	Taken from Ayotte-Beaudet et al.	Teacher	•		
**Neighbourhood greenness**	Normalized Difference Vegetation Index	CANUE[a]	•		
**Child disability status**	Diagnoses of disability, adjustment and learning disabilities	Teacher	•		
**Sex**	Male, female	Student	•		
**School Socioeconomic status**	Ministry of Education	MEQ[b]	•		

a. CANUE: Canadian Urban Environmental Health Research Consortium
b. MEQ: Ministère de l’Éducation du Québec / Québec Ministry of Education

5.1. Primary outcome: Mental health symptoms

The Social Behavior Questionnaire[29] is a 30 item questionnaire that will be used to assess a range of mental health symptoms in children. The instrument incorporates items adapted from the Child Behavior Checklist,[30] the Ontario Child Health Study Scales[31] and the Preschool Behavior Questionnaire[32] used in the Quebec Longitudinal Study of Child Development.[33] The frequency of

children's symptoms over the last 2 months is rated on a 3-point scale (never/not true = 0, sometimes/somewhat true = 1, often/very true = 2). Overall symptoms will be examined as outcomes, as well as internalizing symptoms (emotional distress and withdrawal; 11 items), externalizing symptoms (impulsive/hyperactive/inattentive and disruptive behaviors; 13 items), and social behaviors (pro-social behavior and peer relationships; 6 items).[34] Ratings will be obtained by both child and teacher reports which will be analyzed separately.

5.2. Secondary outcomes

Additional mental health indicators for children. The Positive and Negative Affect Schedule for Child (PANAS-C)[35] is a 20-item scale that will be used to assess positive (e.g., "excited") and negative (e.g., "upset") affect which has good convergent and discriminate validity among children. Children will indicate to what extent they experience feelings over the last 2 weeks (1 = very slightly or not at all; 5 = extremely). The Children's Depression Inventory-Short Version (CDI-S)[36] includes 13 items that will be used to assess cognitive, affective and behavioral signs of depression in children. The CDI-S has good convergent, discriminate and factorial validity among children. Children consider how they were feeling over the last 2 weeks and respond on a 3-point scale (e.g. 1 = I hate myself; 2 = I don't like myself; 3 = I like myself).

Connection with nature for children. The Nature Connection Index (NCI)[37] is a self-report questionnaire including 6 items that will be used to assess connectedness to nature. The scale has good validity and reliability among children. Participants respond to affirmations (e.g., nature always makes me happy) using a 7-point scale (1 = strongly agree; 7 = strongly disagree).

Pro-environmental behaviors will be measured by a brief 6-item questionnaire developed in a recent study,[38] that asks children to report the frequency they made an effort to conserve water, energy and recycle trash (1 = always; 5 = never) and the extent to which they agree that they are ready to volunteer, give money and talk to their entourage to protect nature (1 = strongly agree; 4 = strongly disagree). Additionally children will be asked about their pro-environmental behaviors in the context of climate change using 4 self-report questions, adapted from a previous study and refined by experts.[39,40] Children report the extent to which they are worried about climate change (1 = not at all worried; 4 = extremely worried), that these worries motivate them to engage in pro-environmental behaviors or activism (1 = not at all; 4 = a lot) and that they feel capable of making behavioral changes to help the environment (1 = strongly disagree; 4 = strongly agree).

Well-being for teachers. The World Health Organization Well-Being Index (WHO-5)[41] is a 5-item self-report measure that will assess of wellbeing for adults. The WHO-5 is a widely used, valid and reliable measure that is sensitive to change. Participants indicate the frequency of their feelings (e.g. in a good mood) over the last two weeks (5 = all the time; 0 = never). The Positive and Negative Affect Schedule (PANAS)[42] is a 20-item self-report measure that will be used to assess positive (e.g., "excited") and negative (e.g., "upset") affect which has well-established good psychometric properties. Teachers will indicate to what extent they experience feelings over the last two weeks (1 = very slightly or not at all; 5 = extremely).

Appreciation of outdoor activities for children and teachers. An adapted version of the Enjoyment of Teaching Mathematics Scale[43] will be used for teachers from the intervention group at post-test to self-report their enjoyment of teaching outdoors (e.g. I really like teaching outdoors) with 5 items on a 5 point scale (1 = strongly agree; 5 = strongly disagree). We designed one item for children to self-report the frequency that they appreciated the intervention (1 = not at all; 4 = always) that will be administered in the intervention group at post-test.

5.3. Moderators and descriptive variables

Sex. The children's sex (male, female) will be self-reported by students and additionally reported by teachers.

Disability status of children. Children formally diagnosed as having a physical or mental disability (e.g., intellectual deficiency) or having adaptation disorders (e.g., conduct disorders) or learning disorders (e.g., language deficits) will be identified by teachers.

Greenness of neighbourhoods. The Normalized Difference Vegetation Index (NDVI) will be used to quantify the density of green vegetation associated with the school's zip code. The widely used NDVI is based on the land surface reflectance of colors which is drawn from satellite images of the earth's surface. The index varies between + 1 and -1 with higher values indicating higher green vegetation density. We will use 2019 satellite images which are available via the Canadian Urban Environmental Health Research Consortium (CANUE).[44]

Deprivation indicator of the school. School's level of deprivation will be quantified using a ranking provided by the Ministry of Education and Higher Education. The ranking is based on a composite score incorporating the proportion of students within each school whose mother completed high-school and whose both parents are employed full time. Schools are classified on a scale ranging from 1 (lowest deprivation) to 10 (highest deprivation).[45]

Experience with outdoor teaching of teachers. Teachers' experience with outdoor teaching over the last 3 years (e.g., context, duration) (e.g., did you practice outdoor education in autumn 2022?) and outdoor activities practiced in their leisure time will be assessed with a 6 questions that were successfully used in a previous study on school based outdoor education.

5.4. Exploratory variables (measured only at one time point and/or not used as indicators of the effectiveness of the intervention)

Self-harm. Self-harm will be self-reported by the child at T2 using the Non-Suicidal Self-Injury (NSSI) subscale of the Self-Harm Inventory.[46–48] Students will be asked if they ever intentionally engaged in five self-harm behaviors (cutting, burning, hitting, head banging, scratching) (1=yes, 0=no). If a participant answers yes, they will be prompted to report how many times they have engaged in the behavior. The instrument yields two scores: engagement in any self-harm behavior (1=yes, 0=no) and the number of self-harm behaviors reported (continuous).

Screen time. Screen time will be self-reported by the child at T3 using 6 items drawn from the Québec Health Survey of High School Students (QHSHSS).[49] The QHSHSS has been successfully used in previous surveys for same aged children from the Direction régionale de santé publique. Students will be asked to report how often they engage in passive screen use (e.g. watching series or movies) and active screen use (e.g. video games, chatting, surfing internet) on weekends and on weekdays on a response scale ranging from 0=never to 7=more than 4 hours per day. The instrument yields two scores: engagement in more than the recommended two hours of screen use on weekdays and on the weekend (1=yes, 0=no).

Eco-anxiety. Additionally children will be asked about their eco-anxiety in the context of climate change using 4 self-report questions, adapted from a previous study and refined by experts.[39,40] Children will self-report at all time points the extent to which they are worried about climate change (1=not at all worried; 4=extremely worried), that these worries motivate them to engage in pro-environmental behaviors or activism (1=not at all; 4=a lot) and that they feel capable of making behavioral changes to help the environment (1=strongly disagree; 4=strongly agree).

<u>6. Description of the intervention</u>

6.1. Eligibility criteria

Participating schools will be recruited from a list of 280 French language elementary schools in the Canadian province of Quebec, enrolled in a larger study conducted by the Observatoire pour

l'Éducation et la Santé des Enfants (OPES), which aims to document the consequences of the COVID-19 pandemic on 4th grade children's academic achievement (www.observatoireenfants.ca/en). Additional inclusion criteria for schools are: (a) approval by their school board, (b) access to a greenspace / natural environment (e.g., park, wooded area) on school grounds or within 1 kilometer of the school and (c) having 5-6th grade teachers provide informed and written consent to participate in this study. Inclusion criteria for children to participate in assessments are (a) enrollment in 5-6th grade, (b) providing their assent and (c) having their parents or legal guardians provide informed written consent. As indicated on the information and consent form, participants can withdraw from the study at any time.

6.2. Intervention group

The intervention aims to provide approximately 2 hours of exposure to nature per week, in addition to allowing teachers to access a toolkit of pedagogical and mental health activities grounded in positive psychology. The rationale for *Open Sky School* was informed by the growing literature on the benefits of spending time in nature (greenspace) for mental health.[7] For our intervention, exposure to nature consists of teachers bringing their students to the highest quality greenspace within 1 kilometer of their classroom, which could be located on or off campus. For instance, the location could be a forest on campus or a park nearby. Classes will spend a total of 2 hours (i.e. 2 one hour visits or one 2 hour visits) per week for 12 weeks (transportation time included). The exposure time was chosen in line with recent guidelines recommending a minimum of 2 hours per week in nature.[44]

The main component of the *Open Sky School* consists of exposing children to nature. The nature exposure embeds various activities (academic and mental health components), while providing teacher training and support. To this end, we designed an online toolkit of pedagogical and mental health activities that can be conducted outdoors. The mental health activities are rooted in positive psychology (mindfulness, philosophy for children and art therapy) and are designed to improve children's mental health.[45] The pedagogical activities (French language, mathematics and sciences) are aligned on the core academic competencies required by the Ministry of Education of Québec. Teachers will also be allowed to use their own pedagogical activities (if they do, they will be asked to describe their activities), but are encouraged to use those provided in the toolkit, because they were professionally designed and positively appraised in the quality assessment phase. Nevertheless, as our trial primarily aims to reduce mental health problems among children, teachers will be asked to carry out at least 10 mental health activities provided in the toolkit. The toolkit also includes brief video-based modules on best practices and tips for implementing mental health activities outdoors (i.e., how to deal with negative emotions; how children can remain mindful in a noisy environment). Our experts and graduate students in education and clinical psychology will provide teachers with up to 1 hour per week of virtual optional consultation during the intervention to discuss any arising issues related to the intervention and its implementation. Licensed psychologists from the research team will offer psychological support to any participant who reports high levels of psychological distress and reorientate them to appropriate services if needed.

6.3. Control group

Six months after the start of the trial, elementary school students (and their teachers) in the control group will receive an unguided version of the intervention, supplemented by an online peer support group. As a significant amount of children in the control group will be in secondary school with different teachers by then (i.e., those who were in 6th grade), we will provide these students with a toolkit of 10 mental health activities, adapted from the activities provided to teachers, that they can practice alone, in addition to support via video-conference, phone or email if they require help from a member of the research team while practicing the activities. As in the intervention group, we will

provide support by licensed psychologists at the request of any child, if for example a child reports high levels of psychological distress, and orient children to appropriate services if needed.

7. Implementation of the intervention (fidelity)

The teachers will fill out an online logbook in which they will indicate information about each nature visit. They will provide information on the activities they chose to carry out (from our toolkit or others of their choice), the duration of these activities, where the activity was carried out and the total time spent outdoors. They will record if they watched our training videos, if they encountered any problems and the amount of time they spent with our supervisors. This information will be used to evaluate the adequacy of the implementation of our intervention. To fully comply with implementation, there must be of a total of 24 h of intervention (2 h per week for 12 weeks) and a minimum of 10 mental health activities from the toolkit must be carried out. Classes that do not achieve a minimum of 80% completion and comply with content 90% of the time will be excluded from sensitivity analyses. Information contained in the logbooks will be evaluated independently by two researchers and we will analyze inter-rater agreement to ensure the consistency of the codification of the information provided.

8. Statistical analyses and power

All analyses will be conducted at the cluster level, i.e., at the school level. Dependent variables will be measured on a continuous scale. Child mental health will be assessed at T1, T2, T3. A longitudinal ANCOVA, as described in Liu et al (2009),[50] will be used to estimate the main and moderating effects described above. The characteristics of the intervention group and the control group will be compared at the class level, where means (e.g., socioeconomic deprivation) could be added to the model as covariates, in the unlikely event that randomization would not have balanced the two groups. A total of 80 to 100 schools (with a minimum of 40 classes if only one class per school participates) will detect small effects (power >90%). An intention-to-treat analysis approach will be used to increase the internal and external validity of the project. The analyses will include any child for whom at least one questionnaire has been completed at least one measurement time and the multiple imputations will be used in case of significant loss to follow-up.

9. Preparatory and follow-up studies

9.1. Evaluation of the quality of mental health and pedagogical activities in the tool kit

We will recruit 5th and 6th grade teachers to evaluate the quality of 15 wellbeing activities and 14 educational activities involving nature between May and June 2022. These teachers will be recruited via social networks. Approximately 30 teachers will be asked to test 4 activities with their students and answer a short 7-item confidential online questionnaire (i.e., “I would use this activity with another class in the future” using a scale ranging from 1 (strongly disagree) to 5 (strongly agreed)). Teachers will receive a $25 CAD e-transfer as compensation. Teachers will also be invited to provide suggestions to improve activities using an open response format. Consent will be obtained for all teachers (available on demand).

9.2. Pilot

We will recruit teachers from three to six 6th grade classes by sourcing our network. The teachers will participate in a pilot study that will take place from September to December 2022. Teachers and parents or legal guardians of the students will sign information and consent forms (available in French on demand), but will not be randomized. All participants will receive intervention, i.e., access to activities and supervision. The procedure will take place over a shorter period of time (6 weeks instead of 12 weeks). Teachers will receive a financial compensation of $100 CAD per questionnaire ($200 CAD in total) in the form of a gift card. Consent will be obtained for all teachers (available on demand).

9.3. Feedback survey following RCT

We will administer an optional, anonymous survey to teachers who participated in the 12-week intervention program as part of the randomized controlled trial. The goal of this survey is to obtain feedback on teachers' experience during their participation in the intervention and will allow us to better assess the feasibility and effectiveness of *Open sky School* in schools. This feedback will also allow us to modify and improve this curriculum in the future, to better meet the needs of teachers and students. In the fall of 2023, participating teachers in the intervention group will be contacted by email and will be invited to complete the optional survey if they wish to provide feedback on their experience. In winter 2024, participating teachers in the control group who choose to participate in the 12-week intervention will also be contacted by email. The survey will be administered through Qualtrics, a secure online platform. Consent will be obtained for all teachers (available on demand).

References

1. Loose T, Côté S, Malboeuf-Hurtubise C, et al. Protocol for the Open Sky School: a two-arm clustered randomized controlled trial to test the effectiveness of a nature-based intervention on mental health of elementary school children. *BMC Public Health*. 2023;23(1):236. doi:10.1186/s12889-023-15033-y

2. Hall S, Moskovitz C, Pemberton M. Understanding text recycling: a guide for researchers. Text Recycling Research Project. June 2021. textrecycling.org

3. Power C, Kuh D, Morton S. From developmental origins of adult disease to life course research on adult disease and aging: insights from birth cohort studies. *Annu Rev Public Health*. 2013;34:7-28. doi:10.1146/annurev-publhealth-031912-114423

4. Commisso M, Temcheff C, Orri M, et al. Childhood externalizing, internalizing and comorbid problems: distinguishing young adults who think about suicide from those who attempt suicide. *Psychol Med*. Published online June 29, 2021:1-8. doi:10.1017/S0033291721002464

5. Chadi N, Spinoso Di Piano C, Osmanlliou E, Gravel J, Drouin O. Mental health-related emergency department visits in adolescents before and during the COVID-19 pandemic: a multicentric retrospective study. *Journal of Adolescent Health*. Published online Under review.

6. Li W, Wang Z, Wang G, et al. Socioeconomic inequality in child mental health during the COVID-19 pandemic: first evidence from China. *J Affect Disord*. 2021;287:8-14. doi:10.1016/j.jad.2021.03.009

7. Singh S, Roy D, Sinha K, Parveen S, Sharma G, Joshi G. Impact of COVID-19 and lockdown on mental health of children and adolescents: a narrative review with recommendations. *Psychiatry Res*. 2020;293:113429. doi:10.1016/j.psychres.2020.113429

8. Watkins-Martin K, Devantoy A, Bolanis B, et al. The immediate and short-term effects of nature walks on affect and depressive symptoms in adults with clinical depression. In preparation. *In preparation*.

9. Bratman GN, Anderson CB, Berman MG, et al. Nature and mental health: an ecosystem service perspective. *Science Advances*. 2019;5(7):eaax0903. doi:10.1126/sciadv.aax0903

10. Jackson SB, Stevenson KT, Larson LR, Peterson MN, Seekamp E. Outdoor activity participation improves adolescents' mental health and well-being during the COVID-19 pandemic. *International Journal of Environmental Research and Public Health*. 2021;18(5):2506. doi:10.3390/ijerph18052506

11. Engemann K, Pedersen CB, Arge L, Tsirogiannis C, Mortensen PB, Svenning JC. Residential green space in childhood is associated with lower risk of psychiatric disorders from adolescence into adulthood. *PNAS*. 2019;116(11):5188-5193.

12. Wang P, Meng YY, Lam V, Ponce N. Green space and serious psychological distress among adults and teens: a population-based study in California. *Health Place*. 2019;56:184-190. doi:10.1016/j.healthplace.2019.02.002

13. Donovan GH, Michael YL, Gatziolis D, Mannetje A 't, Douwes J. Association between exposure to the natural environment, rurality, and attention-deficit hyperactivity disorder in children in New Zealand: a linkage study. *Lancet Planet Health*. 2019;3(5):e226-e234. doi:10.1016/S2542-5196(19)30070-1

14. Mitchell R. Is physical activity in natural environments better for mental health than physical activity in other environments? *Soc Sci Med*. 2013;91:130-134. doi:10.1016/j.socscimed.2012.04.012

15. Bolanis D, Orri M, Vergunst F, et al. Increased urban greenspace in childhood associated with lower inattention deficit among adolescents. *Social Psychiatry and Psychiatric Epidemiology*. 2024;59(6):947-956. doi:10.1007/s00127-023-02575-0

16. Kotera Y, Lyons M, Vione KC, Norton B. Effect of nature walks on depression and anxiety: a systematic review. *Sustainability*. 2021;13(7):4015. doi:10.3390/su13074015

17. Kotera Y, Richardson M, Sheffield D. Effects of Shinrin-Yoku (forest bathing) and nature therapy on mental health: a systematic review and meta-analysis. *Int J Ment Health Addiction*. Published online July 28, 2020. doi:10.1007/s11469-020-00363-4

18. Song C, Ikei H, Igarashi M, Takagaki M, Miyazaki Y. Physiological and psychological effects of a walk in urban parks in fall. *Int J Environ Res Public Health*. 2015;12(11):14216-14228. doi:10.3390/ijerph121114216

19. Roberts A, Hinds J, Camic PM. Nature activities and wellbeing in children and young people: a systematic literature review. *Journal of Adventure Education and Outdoor Learning*. 2020;20(4):298-318. doi:10.1080/14729679.2019.1660195

20. Faber Taylor A, Kuo FE. Children with attention deficits concentrate better after walk in the park. *J Atten Disord*. 2009;12(5):402-409. doi:10.1177/1087054708323000

21. de Vries S, van Dillen SME, Groenewegen PP, Spreeuwenberg P. Streetscape greenery and health: stress, social cohesion and physical activity as mediators. *Soc Sci Med*. 2013;94:26-33. doi:10.1016/j.socscimed.2013.06.030

22. Richardson EA, Pearce J, Mitchell R, Kingham S. Role of physical activity in the relationship between urban green space and health. *Public Health*. 2013;127(4):318-324. doi:10.1016/j.puhe.2013.01.004

23. Latham RM, Kieling C, Arseneault L, et al. Childhood exposure to ambient air pollution and predicting individual risk of depression onset in UK adolescents. *J Psychiatr Res*. 2021;138:60-67. doi:10.1016/j.jpsychires.2021.03.042

24. Klompmaker JO, Hoek G, Bloemsma LD, et al. Associations of combined exposures to surrounding green, air pollution and traffic noise on mental health. *Environ Int*. 2019;129:525-537. doi:10.1016/j.envint.2019.05.040

25. Sharpe D. Independent thinkers and learners: a critical evaluation of the 'Growing Together Schools Programme.' *Pastoral Care in Education*. 2014;32(3):197-207. doi:10.1080/02643944.2014.940551

26. Romar JE, Enqvist I, Kulmala J, Kallio J, Tammelin T. Physical activity and sedentary behaviour during outdoor learning and traditional indoor school days among Finnish primary school students. *Journal of Adventure Education and Outdoor Learning*. 2019;19(1):28-42. doi:10.1080/14729679.2018.1488594

27. Bølling M, Niclasen J, Bentsen P, Nielsen G. Association of education outside the classroom and pupils’ psychosocial well-being: results from a school year implementation. *J Sch Health*. 2019;89(3):210-218. doi:10.1111/josh.12730

28. Gustafsson PE, Szczepanski A, Nelson N, Gustafsson PA. Effects of an outdoor education intervention on the mental health of schoolchildren. *Journal of Adventure Education and Outdoor Learning*. 2012;12(1):63-79. doi:10.1080/14729679.2010.532994

29. Collet OA, Orri M, Tremblay RE, Boivin M, Côté SM. Psychometric properties of the Social Behavior Questionnaire (SBQ) in a longitudinal population-based sample. *International Journal of Behavioral Development*. 2023;47(2):180-189. doi:10.1177/01650254221113472

30. Achenbach TM. *Manual for the Child Behavior Checklist/4-18 and 1991 Profile.*; 1991.

31. Offord DR, Boyle MH, Racine Y. Ontario Child Health Study: correlates of disorder. *Journal of the American Academy of Child & Adolescent Psychiatry*. 1989;28(6):856-860. doi:10.1097/00004583-198911000-00008

32. Behar LB. The Preschool Behavior Questionnaire. *Journal of Abnormal Child Psychology*. 1977;5(3):265-275. doi:10.1007/BF00913697

33. Orri M, Boivin M, Chen C, et al. Cohort profile: Quebec Longitudinal Study of Child Development (QLSCD). *Social Psychiatry and Psychiatric Epidemiology*. 2021;56(5):883-894. doi:10.1007/s00127-020-01972-z

34. Commisso M, Temcheff C, Orri M, et al. Childhood externalizing, internalizing and comorbid problems: distinguishing young adults who think about suicide from those who attempt suicide. *Psychological Medicine*. 2023;53(3):1030-1037. doi:10.1017/S0033291721002464

35. Laurent J, Catanzaro SJ, Joiner Jr. TE, et al. A measure of positive and negative affect for children: scale development and preliminary validation. *Psychological Assessment*. 1999;11(3):326-338. doi:10.1037/1040-3590.11.3.326

36. Ahlen J, Ghaderi A. Evaluation of the Children’s Depression Inventory—Short Version (CDI–S). *Psychological Assessment*. 2017;29(9):1157-1166. doi:10.1037/pas0000419

37. Richardson M, Hunt A, Hinds J, et al. A measure of nature connectedness for children and adults: validation, performance, and insights. *Sustainability*. 2019;11(12). doi:10.3390/su11123250

38. Keith RJ, Given LM, Martin JM, Hochuli DF. Urban children’s connections to nature and environmental behaviors differ with age and gender. *PLOS ONE*. 2021;16(7):e0255421. doi:10.1371/journal.pone.0255421

39. Hickman C, Marks E, Pihkala P, et al. Climate anxiety in children and young people and their beliefs about government responses to climate change: a global survey. *The Lancet Planetary Health*. 2021;5(12):e863-e873. doi:10.1016/S2542-5196(21)00278-3

40. Vergunst F, Berry HL. Climate change and children's mental health: a developmental perspective. *Clinical Psychological Science*. 2022;10(4):767-785. doi:10.1177/21677026211040787

41. Topp CW, Østergaard SD, Søndergaard S, Bech P. The WHO-5 Well-Being Index: a systematic review of the literature. *Psychotherapy and Psychosomatics*. 2015;84(3):167-176. doi:10.1159/000376585

42. Watson D, Clark LA, Tellegen A. Development and validation of brief measures of positive and negative affect: the PANAS scales. *Journal of Personality and Social Psychology*. 1988;54(6):1063-1070. doi:10.1037/0022-3514.54.6.1063

43. Frenzel AC, Goetz T, Lüdtke O, Pekrun R, Sutton RE. Emotional transmission in the classroom: exploring the relationship between teacher and student enjoyment. *Journal of Educational Psychology*. 2009;101(3):705-716. doi:10.1037/a0014695

44. Gorelick N, Hancher M, Dixon M, Ilyushchenko S, Thau D, Moore R. Google Earth Engine: planetary-scale geospatial analysis for everyone. *Remote Sensing of Environment*. 2017;202:18-27. doi:10.1016/j.rse.2017.06.031

45. Riglea T, Kalubi J, Sylvestre MP, et al. Social inequalities in availability of health-promoting interventions in Québec elementary schools. *Health Promotion International*. 2022;37(1):daab023. doi:10.1093/heapro/daab023

46. Eggermont K, Bastin M, Luyckx K, Claes L. Do gender and age moderate the relationship between friendship quality and non-suicidal self-injury in community children and adolescents? *Psychologica Belgica*. Published online 2021. doi:10.5334/pb.1067

47. Sansone RA, Sansone LA. Measuring self-harm behavior with the Self-Harm Inventory. *Psychiatry (Edgmont)*. 2010;7(4):16-20.

48. Thümmler S, Askenazy F. K-SADS-PL DSM-5: French version Mai 2018 (of K-SADS-PL DSM-5 November 2016, Kaufmann J, Birmaher B, Axelson D, Perepletchikova F, Brent D, Ryan N). Published online 2018. http://sfpeada.fr/

49. Institut de la statistique du Québec. Enquête québécoise sur le tabac, l'alcool, la drogue et le jeu chez les élèves du secondaire, 2019. Institut de la statistique du Québec. Accessed September 12, 2024. https://statistique.quebec.ca/fr/document/enquete-quebecoise-tabac-alcool-drogue-jeu-eleves-secondaire-2019

50. Liu X, Wang L. Sample size planning for detecting mediation effects: a power analysis procedure considering uncertainty in effect size estimates. *Multivariate Behav Res*. 2019;54(6):822-839. doi:10.1080/00273171.2019.1593814
