# Supplementary material for: A Nature-Based Intervention and Mental Health of Schoolchildren: A Cluster Randomized Clinical Trial
Source: JAMA Netw Open. 2024 Nov 15;7(11):e2444824. doi: 10.1001/jamanetworkopen.2024.44824 (PMC11568460; doi:10.1001/jamanetworkopen.2024.44824)
Supplement: Supplement 2. — eMethods 1. Deviation From Original Protocol eMethods 2. Description of Measures eMethods 3. Description of the Intervention eMethods 4. Contamination Effects in the Intervention and Control Groups eTable 1. Characteristics of the Intervention Group—Teacher Logbook eTable 2. P Values for the Interactions Between Moderators and Intervention Effectiveness eReferences [file jamanetwopen-e2444824-s002.pdf]

Supplementary Online Content

Loose T, Fuoco J, Malboeuf-Hurtubise C, et al. A nature-based intervention and mental health of school children: a cluster randomized clinical trial. *JAMA Netw Open*. 2024;7(11):e2444824. doi:10.1001/jamanetworkopen.2024.44824

eMethods 1. Deviation From Original Protocol
eMethods 2. Description of Measures
eMethods 3. Description of the Intervention
eMethods 4. Contamination Effects in the Intervention and Control Groups
eTable 1. Characteristics of the Intervention Group—Teacher Logbook
eTable 2. *P* Values for the Interactions Between Moderators and Intervention Effectiveness
eReferences

This supplementary material has been provided by the authors to give readers additional information about their work.

© 2024 Loose T et al. *JAMA Network Open*.

eMethods 1. Deviations from original protocol

Recruitment
We planned to exclusively recruit from a list of 281 schools enrolled in a larger study (as described above). We began the recruitment process using this list but responses were substantially lower than anticipated. We decided to additionally recruit via social networks to boost participation. Even with this additional recruitment method we were able to recruit 1000 students rather than the anticipated 2500. Common reasons that teachers declined to participate included (a) not wanting to add strain to the curriculum because of a standardized exam at the end of 6th grade, (b) difficulty with obtaining parental consent forms and (c) not wanting to add to their workload.

Measures
Justification for primary outcome measure: Previous studies suggest that greenspace exposure can have differential associations with internalizing and externalizing symptoms among youth. As such, we retained the three subscales of the Social Behaviour Questionnaire, namely internalizing symptoms, externalizing symptoms and social problems and omit the overall mental health score.

Statistical analyses
After consulting with a statistician, we revised the analyses from a longitudinal ANCOVA to a clustered mixed model. This change is better justified given that our design only includes measures at baseline and immediate follow-up, rather than the originally planned three time points.

We also added post-hoc moderation analyses by baseline levels of mental health symptoms. We observed low levels of mental health symptoms in our student sample and variability was low. As such, we realized that the intervention could not decrease problems that were at most often at low levels or non-existent.

© 2024 Loose T et al. *JAMA Network Open*.

eMethods 2. Description of measures

Primary outcome: Children's mental health

The Social Behavior Questionnaire (SBQ[1]; 30 items) was used to evaluate mental health among children. The SBQ evaluates internalizing symptoms (2 subscales: emotional distress and withdrawal), externalizing symptoms (2 subscales: impulsive/hyperactive/inattentive and disruptive behaviors), and social problems (2 subscales: prosocial behavior and peer relationships).[2] Teachers reported on the frequency of children's symptoms over the last 2 months on a 3-point scale (never/not true = 0, sometimes/somewhat true = 1, often/very true = 2). We used a parallel version of the SBQ for children to self-report their mental health symptoms. The psychometric properties of the child-report version have not been formally studied, but the items have been used in previous studies. Cronbach's alpha are as follows. For child report at baseline: α=.76 for internalizing symptoms (11 items), α=.81 for externalizing symptoms (13 items) and α=.66 for social problems (6 items). For child report at follow-up: α=.78 for internalizing symptoms, α=.82 for externalizing symptoms and α=.68 for social problems. For teacher report at baseline: α=.83 for internalizing symptoms, α=.90 for externalizing symptoms and α=.70 for social problems. For teacher report at follow-up: α=.81 for internalizing symptoms, α=.89 for externalizing symptoms and α=.66 for social problems.

Secondary Outcomes

Additional mental health indicators for children

The Positive and Negative Affect Schedule for Child (PANAS-C)[3] is a 20-item scale that was used to assess positive (e.g., "excited") and negative (e.g., "upset") affect, which has good convergent and discriminate validity among children. Children indicated to what extent they experienced feelings over the last 2 weeks (1 = very slightly or not at all; 5 = extremely). Cronbach's alpha are as follows: baseline: α=.80 for positive affect, α=.83 for negative affect; follow-up: α=.82 for positive affect, α=.87 for negative affect.

The Children's Depression Inventory-Short Version (CDI-S)[4] includes 13 items and was used to assess cognitive, affective and behavioral signs of depression in children. The CDI-S has good convergent, discriminate and factorial validity among children. Children considered how they were feeling over the last 2 weeks and responded on a 3-point scale (e.g. 1 = I hate myself; 2 = I don't like myself; 3 = I like myself). Cronbach's alpha are as follows: baseline: α=.85; follow-up: α=.88.

Relationship with environment for children

The Nature Connection Index (NCI)[5] is a self-report questionnaire including 6 items that was used to assess connectedness to nature. The scale has good validity and reliability among children. Children responded to affirmations (e.g. nature always makes me happy) using a 7-point scale (1 = strongly agree; 7 = strongly disagree). Cronbach's alpha are as follows: baseline: α=.89; follow-up: α=.91.

© 2024 Loose T et al. *JAMA Network Open*.

Pro-environmental attitudes and efforts were measured by a brief 6-item questionnaire developed in a recent study,[6] that asks children to report the frequency they made an effort to conserve water, energy and recycle trash (1 = always; 5 = never) and the extent to which they agree that they are ready to volunteer, give money and talk to their entourage to protect nature (1 = strongly agree; 4 = strongly disagree). Cronbach's alpha are as follows: baseline: α=.59 for environmental attitudes, α=.73 for environmental efforts; follow-up: α=.67 for environmental attitudes, α=.78 for environmental efforts.

Moderator variables

Sex

The children's sex (male, female) was reported by the teacher and self-reported by the child.

Disability status of children

Children formally diagnosed as having a physical or mental disability (e.g. intellectual deficiency) or having adaptation disorders (e.g. conduct disorders) or learning disorders (e.g. language deficits) were identified by teacher reports.

Greenspace of neighbourhoods

The Normalized Difference Vegetation Index (NDVI) was used to quantify the density of green vegetation associated with the school's postal code. The widely used NDVI is based on the land surface reflectance of colors which is drawn from satellite images of the earth's surface. The index varies between + 1 and -1 with higher values indicating higher green vegetation density. We used 2019 satellite images which are available via the Consortium CANUE.[7] 250m was used.

School socioeconomic disadvantage of the school

School's *socioeconomic disadvantage* was quantified using a ranking provided by the Ministry of Education and Higher Education. The ranking is based on a composite score incorporating the proportion of students within each school whose mothers completed high-school and whose both parents are employed full time. Schools were classified on a scale ranging from 1 (lowest deprivation) to 10 (highest deprivation) and scores of 8 to 10 are considered as disadvantaged.[8] The 2 private schools in our sample were assigned a score of 1 (lowest deprivation).

Experience with outdoor teaching of teachers

We assessed teacher's outdoor teaching experience over the last 3 years (1=yes, 0=no).

Teacher log book

In the intervention group, teachers reported on the characteristics of their outdoor interventions on a weekly basis. Each entry inquired about: the number of outings completed during a given

© 2024 Loose T et al. *JAMA Network Open*.

week; the total time spent outdoors (transportation time included); the location(s) of their outing(s) (i.e., forest or wooded area, school yard, park, garden, other); the types of activities they selected (i.e., from the toolkit vs. their personal activities); whether consultation from a team member was requested and the type of consultation (i.e., individual or group meeting); as well as any comments about facilitating conditions or challenges they experienced during their time spent outdoors for the given week.

© 2024 Loose T et al. *JAMA Network Open*.

eMethods 3. Description of the intervention

The rationale for Open Sky School was informed by the growing literature on the benefits of spending time in nature for mental health.[9] Our intervention aimed to provide approximately 120 minutes of exposure to nature each week over 12 weeks, with teachers bringing their students to the highest quality green space within 1 km of their school (on or off school grounds). This could include a forest on school grounds or a nearby park. The prescribed exposure involved either two one-hour visits or one two-hour visit per week, transportation time included, aligning with recent guidelines recommending a minimum of 2 hours per week in nature.[10] In Open Sky School, the primary element involved exposing children to nature through diverse activities, supported by teacher training and assistance.

To this end, we designed an online toolkit of pedagogical (language arts, mathematics, and sciences) and mental health activities (mindfulness, philosophy for children, and art therapy)[11] accessible on our website : https://www.ecoleacielouvert.ca/en/. Teachers were allowed to use their own pedagogical activities (if they did, they were asked to describe their activities on a weekly basis), but were encouraged to use those provided in the toolkit, as they were professionally designed and positively appraised in a previous quality assessment phase. Nevertheless, as our trial primarily aimed to reduce mental health problems among children, we requested that teachers carry out at least 10 mental health activities provided in the toolkit. As art therapy, philosophy for children and mindfulness are not usually taught in elementary school, the toolkit also included three brief video-based modules on best practices for implementing mental health activities outdoors (i.e., how to manage negative emotions; how children can remain mindful in a noisy environment). Our experts and graduate students in education and clinical psychology provided teachers with up to 2 hours per week of virtual optional drop-in consultation during the first 3 weeks of intervention to discuss any arising questions or issues related to the intervention and its implementation, with other participating teachers present. From the fourth to the final week of intervention, members of the research team were available to answer any teachers' questions by email, phone, or virtual appointment, upon request. Note that very few teachers solicited our services. Licensed psychologists from the research team offered psychological support to any participant who reported high levels of psychological distress and orientated them to appropriate services if needed.

© 2024 Loose T et al. *JAMA Network Open*.

eMethods 4. Contamination effects in the intervention and control groups

Treatment compliance in the intervention group

We calculated the total number of hours in which classes in the intervention group engaged in outdoor activities over the intervention period (range: 13 to 43.33; M=23.51; SD=7.21). Teachers were asked to conduct 10 mental health activities, but compliance was low (range 0 to 15 activities; M=7.38; SD=3.60). More specifically, 6/26 (23%) teachers achieved 100% compliance (≥ 10 activities) and 12/26 (46%) teachers achieved 80% compliance (≥ 8 activities).

Engagement in outdoor education in the control group

In the control group, we could not prohibit teachers from engaging in outdoor education. We suspect that teachers who agreed to participate in the study were motivated to engage in outdoor education over the intervention period. They signed up before knowing if they would be in the control or intervention group. When assigned to the control group, half of these teachers (13/27) engaged in outdoor education over the intervention period. We judged case by case if the activities of the control group could be comparable to the intervention group. We received emails from participants in the control group expressing disappointment in not being assigned to the intervention group and motivation to engage in outdoor sessions regardless using their own teaching materials. Further, though the intervention group was encouraged to use activities from the tool kit, many teachers in the intervention group opted to use their own activities, which furthers the contamination issue across groups.

At immediate follow-up, we evaluated the frequency teachers engaged in outdoor education. First, teachers indicated if they engaged in any outdoor activities over the intervention period (yes, no). If they indicated yes, they were prompted to provide further details. Using a 4x5 cross table, teachers indicated the frequency at which they engaged in outdoor education: (a) 1 to 60 minutes (b) 61 to 120 minutes (c) half day (d) full day, using a response scale 1=never; 2=one to three times a month; 3=once per week; 4=two to three times per week; 5=four to five times per week. We also asked where they conducted outdoor activities using check boxes for the following options: school yard, outdoor classroom, woods or forest, municipal park, lake or pond, neighborhood street, sports complex or other.

We used this information to approximate the amount of time that the control group engaged in outdoor teaching activities over the intervention period. The following scoring was used:

Time: For the time interval response options, we retained the mid-point (1 to 60 minutes=30 minutes; 61-120 minutes=90 minutes). For half days or full days, we retained the value of 150 minutes (midpoint between 121-180).

Frequency: For the time interval response options, never=0 times per month; 1-3 times per month=2 times per month; once a week= 4 times per month. No teachers chose the response options 2-3 times per week or 4-5 times per week.

Location: Teachers who did not tick a box including a green space were excluded.

© 2024 Loose T et al. *JAMA Network Open*.

eTable 1. Characteristics of the intervention group: Teacher logbook

**Intervention group characteristics**	**M**	**SD**	**Min**	**Max**
Total weeks engaged in outdoor activities	10.96	0.94	9	12
Total number of outings	20.42	4.95	11	34
Time in minutes per week outdoors	115.98	33.76	65	216.67
**Total number of activities from toolkit**				
Total pedagogical activities	5.28	3.56	0	13
French language	2.31	1.58	0	5
Mathematics	1.14	1.03	0	3
Science	1.82	1.78	0	5
Total wellbeing activities	7.54	3.24	0	15
Art therapy	2.52	1.83	0	6
Mindfulness	3.46	2.27	0	8
Philosophy	1.56	1.94	0	11
Total other activities	11.17	5.51	2	21
**Number of outings by location**				
School yard	3.81	2.22	0	8
Park	3.42	3.14	0	11
Wooded area	2.89	3.45	0	11
Garden	0.78	1.82	0	8
Other	2.04	2.26	0	8

© 2024 Loose T et al. *JAMA Network Open*.

eTable 2. P values for the interactions between moderators and intervention effectiveness

	Child's Sex	Disability status [a]	NDVI [b]	School's Socioeconomic Disadvantage [c]	Outdoor teaching experience [d]
			P-values		
**Teacher report**					
Internalizing	.03	.41	.66	.15	.19
Externalizing	.30	.17	.15	.90	.07
Social problems	.77	.10	.54	.84	.17
**Student report**					
Internalizing	.45	.65	.15	.62	.78
Externalizing	.52	.69	.47	.73	.81
Social problems	.71	.31	.49	.56	.73

a. Teachers reported if the student was in any one of the three following categories established by the Ministry of Education: a) The student presents vulnerability factors likely to influence their learning or their behavior and may thus be at risk, if a rapid intervention is not carried out. This includes students who do not have an official diagnostic code. b) Students with disabilities or social maladjustments or learning difficulties with an official diagnostic code. c) Students with social maladjustments or learning difficulties (DAA) without a code who benefit from an intervention plan.

b. The Normalized Difference Vegetation Index (NDVI) quantifies the density of green vegetation associated with the school's zip code. The index varies between + 1 and -1 with higher values indicating higher green vegetation density. Information was sourced from Consortium CANUE. 250-meter buffers is reported.

c. Socioeconomic disadvantage indicator of the school is calculated by the proportion of mothers without higher education and proportion of parents who are unemployed.

d. Teachers reported if they practiced outdoor education over the last three years

© 2024 Loose T et al. *JAMA Network Open*.

eReferences

1. Collet OA, Orri M, Tremblay RE, Boivin M, Côté SM. Psychometric properties of the Social Behavior Questionnaire (SBQ) in a longitudinal population-based sample. *Int J Behav Dev*. 2023;47(2):180-189. doi:10.1177/01650254221113472

2. Commisso M, Temcheff C, Orri M, et al. Childhood externalizing, internalizing and comorbid problems: distinguishing young adults who think about suicide from those who attempt suicide. *Psychol Med*. 2023;53(3):1030-1037. doi:10.1017/S0033291721002464

3. Laurent J, Catanzaro SJ, Joiner Jr. TE, et al. A measure of positive and negative affect for children: scale development and preliminary validation. *Psychol Assess*. 1999;11(3):326-338. doi:10.1037/1040-3590.11.3.326

4. Ahlen J, Ghaderi A. Evaluation of the Children's Depression Inventory—Short Version (CDI–S). *Psychol Assess*. 2017;29(9):1157-1166. doi:10.1037/pas0000419

5. Richardson M, Hunt A, Hinds J, et al. A measure of nature connectedness for children and adults: validation, performance, and insights. *Sustainability*. 2019;11(12). doi:10.3390/su11123250

6. Keith RJ, Given LM, Martin JM, Hochuli DF. Urban children's connections to nature and environmental behaviors differ with age and gender. *PLOS ONE*. 2021;16(7):e0255421. doi:10.1371/journal.pone.0255421

7. Gorelick N, Hancher M, Dixon M, Ilyushchenko S, Thau D, Moore R. Google Earth Engine: planetary-scale geospatial analysis for everyone. *Big Remote Sensed Data Tools Appl Exp*. 2017;202:18-27. doi:10.1016/j.rse.2017.06.031

8. Riglea T, Kalubi J, Sylvestre MP, et al. Social inequalities in availability of health-promoting interventions in Québec elementary schools. *Health Promot Int*. 2022;37(1):daab023. doi:10.1093/heapro/daab023

9. Bratman GN, Anderson CB, Berman MG, et al. Nature and mental health: an ecosystem service perspective. *Sci Adv*. 5(7):eaax0903. doi:10.1126/sciadv.aax0903

10. White MP, Alcock I, Grellier J, et al. Spending at least 120 minutes a week in nature is associated with good health and wellbeing. *Sci Rep*. 2019;9(1):7730. doi:10.1038/s41598-019-44097-3

11. Malboeuf-Hurtubise C, Léger-Goodes T, Mageau GA, et al. Online art therapy in elementary schools during COVID-19: results from a randomized cluster pilot and feasibility study and impact on mental health. *Child Adolesc Psychiatry Ment Health*. 2021;15(1):15. doi:10.1186/s13034-021-00367-5

© 2024 Loose T et al. *JAMA Network Open*.
