# Supplementary material for: A Nature-Based Intervention and Mental Health of Schoolchildren: A Cluster Randomized Clinical Trial
Source: JAMA Netw Open. 2024 Nov 15;7(11):e2444824. doi: 10.1001/jamanetworkopen.2024.44824 (PMC11568460; doi:10.1001/jamanetworkopen.2024.44824)
Supplement: Supplement 3. — Data Sharing Statement [file jamanetwopen-e2444824-s003.pdf]

Data Sharing Statement

Loose. A Nature-Based Intervention and Mental Health of School Children. *JAMA Netw Open*. Published November 15, 2024. doi:10.1001/jamanetworkopen.2024.44824

Data
Additional Information: clinicaltrials.gov, NCT05662436: https://clinicaltrials.gov/study/NCT05662436?term=open%20sky%20school&rank=1#study-record-dates.
Data available: Yes
Data types: Deidentified participant data
How to access data: Data and syntax is available upon reasonable request. marie-claude.geoffroy@mcgill.ca
When available: With publication

Supporting Documents
Document types: None

Additional Information
Who can access the data: researchers whose proposed use of the data has been approved
Types of analyses: Any types
Mechanisms of data availability: after approval of a proposal, and REB approval
